# Restoration of Immune Homeostasis: The Role of miR-30b-5p and Notch Signaling in Uveitis After Treatment With Longdan Xiegan Decoction

**DOI:** 10.1155/mi/8824838

**Published:** 2025-08-28

**Authors:** Yan Qiu, Xuewei Yin, Lijie Guo, Baojian Wang, Miao Zhang, Shuqin Xu, Huixia Wei, Bin Liu, Yane Gao, Hongsheng Bi, Dadong Guo

**Affiliations:** ^1^Department of Rheumatology and Immunology, The Second Affiliated Hospital of Shandong University of Traditional Chinese Medicine, Jinan 250002, China; ^2^Departement of Central Laboratory, Affiliated Eye Hospital of Shandong University of Traditional Chinese Medicine, Jinan 250002, China; ^3^College of Ophthalmology and Optometry, Shandong University of Traditional Chinese Medicine, Jinan 250002, China; ^4^Laboratory Center, Shandong Academy of Eye Disease Prevention and Therapy, Jinan 250002, China; ^5^School of Basic Medical Sciences, Guangzhou Medical University, Guangzhou 510000, China; ^6^Department of Orthopedics, The Third Affiliated Hospital of Beijing University of Chinese Medicine, Beijing 10029, China; ^7^The Second Clinical Medical College, Shandong University of Traditional Chinese Medicine, Jinan 250002, China; ^8^Department of Clinical Laboratory, Affiliated Eye Hospital of Shandong University of Traditional Chinese Medicine, Jinan 250002, China; ^9^The First Clinical Medical College, Shandong University of Traditional Chinese Medicine, Jinan 250355, China

**Keywords:** experimental autoimmune uveitis, Longdan Xiegan decoction, miR-30b-5p, Notch signaling pathway, traditional Chinese medicine

## Abstract

Uveitis is an inflammatory eye disease, and Longdan Xiegan Decoction (LXD) has been used to treat uveitis. However, the underlying mechanisms have not fully been addressed. The present study aimed to provide new insights into LXD ameliorating inflammatory response of experimental autoimmune uveitis (EAU) and regulating T helper (Th) cell differentiation via the interaction between microRNA (miRNA) and mRNA. The hub genes, corresponding miRNAs, and bioactive substances from the LXD formula that are involved in ameliorating uveitis were identified through the network pharmacology analysis. We investigated the efficacy of LXD on the pathogenesis of EAU in vivo using the Genesis-D camera and histopathological examination, and confirmed the inhibitory effect of miR-30b-5p on the Notch signaling pathway in vitro. The analysis results indicated that T-cell lineage commitment and the Notch signaling pathway activation are the primary inflammatory process associated with the therapeutic effects of LXD on uveitis. Further predictions revealed the miRNA–mRNA interactions between miR-30b-5p and the mRNAs associated with Notch1 and DLL4. The pathogenesis of uveitis involves elevated Notch1, DLL4, IL-17A, and RORγt, and decreased levels of miR-30b-5p. LXD can enhance miR-30b-5p expression, inhibit Notch signaling activation, and suppress the commitment of uveitogenic T-cell lineages. Additionally, it downregulates proinflammatory cytokines while upregulating antiinflammatory cytokines, thereby promoting the the expansion of Treg and Th2 cell lineages, and restoring the ratios of Th1/Th2 and Th17/Treg cells. Reduced expression of miR-30b-5p contributes to the pathogenesis of uveitis. LXD exhibited an inhibitory effect on Notch signaling activation through the miR-30b-5p upregulation and the regulation of the balance between Th1/Th2 and Th17/Treg cell differentiation, ultimately facilitating the treatment of uveitis.

## 1. Introduction

Uveitis typically refers to a group of inflammatory disorders affecting ocular region that cause swelling and tissue destruction. It leads to 5%–10% of vision impairment worldwide. Among these, upto 35% of patients have severe visual dysfunction or even blindness [1, 2]. Uveitis can be categorized by the anatomical involvement of the eye (anterior, intermediate, posterior, or panuveitis), or etiology (infectious or noninfectious) [3]. Uveitis' etiopathology and pathogenesis are poorly understood in many cases. Corticosteroids are the main agents in the treatment of uveitis, but their long-term application causes serious ocular and systemic side effects [4]. More effective and safer therapies are required worldwide.

Traditional Chinese medicine (TCM) has been widely accepted and applied for a long history of clinical practice due to its effectiveness, availability, and inexpensiveness. In addition, formulas typically exhibit integral therapeutic effects with fewer incidences of adverse effects, for the complex process of their multicomponent derived from natural sources acts on multitarget and multipathway [5]. The TCM academic concept of addressing the dampness and heat on the liver is a noteworthy clinical approach in the treatment of uveitis [6]. Therefore, it is necessary to search for a solution for uveitis in the domain of TCM. Network pharmacology can uncover the underlying immune therapeutic mechanism of TCM in accordance with existing databases from a comprehensive perspective, which simultaneously matches TCM's ascendancies [7].

Longdan Xiegan Decoction (LXD) is well-known for its ancient and modern clinical applications of eliminating the liver's meridian damp–heat and hepatobiliary real fire on inflammation, whose specific symptoms often include eye condition (redness, itching, and pain) [8]. LXD can exhibit antiinflammatory, immunomodulating, antivirus, and hepatoprotective therapeutic benefits in various eye conditions and has been widely accepted in clinical practice by oral administration such as herpes simplex virus keratitis [9] and neuroretinitis [10]. LXD effectively improved patients' vision and reduced uveitis recurrence in prior trials by modulating IL-10 and IL-17 expression in anterior uveitis treatment [11]. Its exact immunomodulatory mechanisms require further clarification.

MicroRNAs (miRNAs) are short noncoding RNAs originated from genomic DNA. They are responsible for regulating posttranscriptional gene expression, which is vital for various cellular processes such as cell growth, tissue differentiation, proliferation, differentiation, inflammation, apoptosis, and autophagy [12–16]. Notably, specific miRNAs modulate T cell differentiation by targeting key transcription factors; for example, miR-155 enhances T helper (Th)17 polarization while miR-17-92 contributes Th1 differentiation [17]. Among these, miR-30b-5p has emerged as a critical regulator of inflammatory cytokine production and inhibitor of inflammatory signaling pathway to promote immune homeostasis [18, 19]. Consistent with this role, our previous study identified significant downregulation of miR-30b-5p in spleen, lymph nodes, and ocular tissues of experimental autoimmune uveitis (EAU) rats, which correlated with exacerbated autophagy and IL-17-driven inflammation [20, 21]. Mechanistically, increased miR-30b-5p overexpressed M2 macrophage-derived exosomes attenuated pyroptosis via NLRP3/IL-1β suppression, highlighting its potential as a therapeutic mediator for TCM-based uveitis immunomodulation [22].

EAU rats act as a reliable tool in elucidating the mechanisms underlying the inflammatory process and serve as a template for the advancement of novel therapies, as it involves a noninfectious and intraocular inflammatory disease that occurs when immune cells react to self-antigens [23]. In our current investigation, we have identified molecules within the Notch signaling pathway as potential therapeutic targets based on network pharmacology predictions and previous studies. Subsequently, we conducted in vivo and in vitro experiments to examine the effects of LXD on the generation of miR-30b-5p. Our aim was to explore the role of miR-30b-5p in inhibitory effect of LXD on Notch signaling activation and LXD's immunomodulatory effects in the treatment of uveitis.

## 2. Materials and Methods

### 2.1. The Identification and Initial Screening Process of Bioactive Components From LXD

We gathered data on compounds in the LXD along with related information, by utilizing sources such as TCM Systems Pharmacology (TCMSP) and the TCM Database Taiwan (http://tcm.cmu.edu.tw/review-result.php?herbid=748) database. The active ingredients in LXD were detected through absorption, distribution, metabolism, and excretion (ADME) by utilizing the TCMSP. In this study, we established selection criteria for active compounds, requiring oral bioavailability (OB) ≥ 30% and drug-likeness (DL) ≥ 0.18 [24]. For the sake of comprehensive pharmacodynamic gene matching, in addition to the main databases such as PubChem, Pharmmapper, and UniProt, we also utilized Swiss Target Prediction to forecast the potential targets of selected components from LXD. Besides the SDF format of 2D structures of active ingredients, the data of 3D chemical structures were also acquired. As molecule probes, the 3D chemical structures' data were input into the Pharmmapper server for more accurate pharmacodynamic gene matching, complemented by the most probable macromolecular targets of its 2D molecule probes estimated through Swiss Target Prediction. UniProt standardized the presumptive therapeutic target information corresponding to active ingredients.

### 2.2. Collection of the Candidate Biomarkers of Uveitis

Uveitis-related targets were extracted through mining the online Mendelian inheritance in man (OMIM) and GeneCards. To discover the candidate biomarkers and potential therapeutic targets among differentially expressed genes (DEGs), the gene expression dataset of GSE37588 obtained from NCBI Gene Expression Omnibus (GEO, http://www.ncbi.nlm.nih.gov/geo/) was processed by the “Limma” package in R.

### 2.3. Network Construction of Drug-Disease Crossover Genes and the Identification of Hub Genes

To dredge up the direct or indirect relationship, we applied String 11.0 database to build the targets' protein–protein interactions (PPIs) with a combined score > 0.9. CytoHubba is a plugin in the Cytoscape platform with 11 scoring algorithms, among which the proposed MCC and DMNC algorithm performs better catching more elemental proteins. Since the biological PPIs are heterogeneous, we adopted MCC and DMNC to evaluate the importance of the nodes in addition to the degree algorithm [25]. In this study, the top 50% of nodes were picked out as hub genes according to each of the result lists and taken the union of them to construct a network.

### 2.4. Bioinformatics Annotation of Drug-Disease Crossover Genes

The MCODE plugin was applied to divide the above network into several modules that consisted of nodes with similar or related functions. We conducted Gene Ontology (GO) (biological process, cellular component, and molecular function annotation) and biological pathways (WikiPathway) enrichment analysis using the metascape (https://metascape.org/gp/index.html#/main/step1). Because EAU is mainly associated with autoimmune factors, we performed GO annotation concentrated on immune system process terms in the ClueGo plugin. Targets within immune system process terms were further used to establish a compound-target-disease (C-T-D) network to find out the bioactive substance of drugs exerting major therapeutic effects on the most crucial contributor to the main disease.

### 2.5. Prediction of Corresponding miRNAs

We used key genes to obtain miRNAs associated with hub genes, which were ranked based on integrative confidence score determined by RNAInter (http://www.rnainter.org/) [26]. MiRPathDB (https://mpd.bioinf.uni-sb.de/heatmap_calculator.html?organism=hsa) provided biochemical annotation of the predicted miRNAs. We employed TargetScan (http://www.targetscan.org/vert_71/) to identify potential target genes that contain binding sites for miR-30b-5p in their 3′ untranslated regions [27].

### 2.6. Animals

The present study has been approved by the Laboratory Animal Management Committee of the Affiliated Hospital of Shandong University of Traditional Chinese Medicine (AWE-2022-017). In the present study, 6–8-week-old female Lewis rats (170 ± 10 g) were purchased from Beijing Vital River Laboratory Animal Co., Ltd. (Beijing, China) and authorized under animal license number SCXK (Jing 2016-0006). Prior to the experiment, the rats were first adapted to a constant temperature and humidity living environment with 12 h light/dark cycles for 1 week.

### 2.7. Reagents

Phosphate-buffered saline (PBS), formaldehyde, paraffin, and hematoxylin and eosin (H&E) were supplied from Sinopharm Chemical Reagent Co., Ltd. (Shanghai, China); FITC-CD4, APC-CD25, PE-IL-17A, PE-Foxp3, PE-IL-4, PE-IFN-γ (Bioss, Beijing, China); complete Freund's adjuvant (CFA) (Sigma, USA); Mycobacterium tuberculin H37RA (TB) (Difco, USA); peptide R16 of bovine interphotoreceptor retinoid-binding protein (IRBP) (residues 1177–1191, sequences: ADGSSWEGVVPDV) (Sangon Biotech., Shanghai, China); BCA Assay Kit and EasyRNA Tissue/Cell Rapid Extraction Kit (Shandong Sparkjade Biotech., Co., Ltd., Jinan China); First Strand cDNA Synthesis Kit (Vazyme Biotech Co., Ltd., Nanjing, China); rat Foxp3/RORγt/Notch1/DLL4/IL-10/IL-17A/IFN-γ/IL-4 ELISA Kits (JL, Jianglai Biological, Shanghai, China); riboFECT CP transfection agent (RiboBio, China).

### 2.8. The Preparation of LXD Extract and Medicated Serum

The LXD extract was boil-free granules, and the composition of the ultimate formulation was composed of *Gentiana scabra Bunge* (*Long Dan* in Chinese) (6 g), *Scutellaria baicalensis Georgi* (*Huang Qin* in Chinese) (9 g), *Gardenia jasminoides J.Ellis* (*Zhi Zi* in Chinese) (9 g), *Alisma plantago-aquatica subsp. orientale* (*Sam*.) *Sam*. (*Ze Xie* in Chinese) (12 g), *Akebia trifoliata* (*Thunb*.) *Koidz*. (*Mu Tong* in Chinese) (9 g), *Plantago asiatica L*. (*Che Qian* in Chinese) (9 g), *Angelica sinensis* (*Oliv*.) *Diels* (*Dang Gui* in Chinese) (8 g), *Rehmannia glutinosa* (*Gaertn*.) *DC*. (*Di Huang* in Chinese) (10 g), *Bupleurum chinense DC*. (*Chai Hu* in Chinese) (10 g), and *Glycyrrhiza glabra L*. (*Gan Cao* in Chinese) (6 g). One dose of boil-free granule was equivalent to 64 g of crude drug, which added boiling water (192 mL) to guarantee the drug concentration of 0.33 g/mL. LXD formula granules (batch number: 1311007W, Resources Sanjiu Pharmaceutical Co., Ltd., Shenzhen, China.) were verified using thin layer chromatography (TLC) in accordance with Chinese Pharmacopeia (edition 2015). All standard reference herbs and reference substances were endorsed by National Institute for the Control of Pharmaceutical and Biological Products, China. The botanical name was validated by consulting the MPNS database (http://mpns.kew.org) on May 6, 2023. The clinical equivalent consistency was converted to five times the dosage when used in rats, and the rats in the LXD group were treated by oral gavage (1000 mg kg^−1^ d^*−*1^) once daily [28]. The clinical equivalent consistency was converted to five times the dosage when used in rats. Ten adult healthy female Lewis rats were randomly divided into two groups (*n* = 5), gavaged by LXD (1000 mg kg^−1^ d^−1^) and sterilized PBS (0.6 mL) once daily for consecutive 5 days, respectively. 5 mL of blood was aseptically drawn from the heart on day 5 to obtain serum. The serum was inactivated at 56°C for 30 min, filtered through a 0.22 μm cellulose acetate membrane, and stored at −80°C until utilized.

### 2.9. The Induction of EAU and Intervention With LXD In Vivo

One hundred healthy female Lewis rats (6–8-week-old) were divided into six groups, that is, a normal control group (NC, *n* = 20), an EAU group, a prednisone acetate group (PA, 6 mg/kg, converted from the clinical dose, *n* = 20), an LXD low dose group (L-LXD, 700 mg/kg, *n* = 20), an LXD medium dose group (M-LXD, 1000 mg/kg, *n* = 20), and an LXD high dose group (H-LXD, 1300 mg/kg, *n* = 20). The IRBP emulsion was created by dissolving 100 μg of IRBP peptide along with 100 μg of TB and 150 μL of CFA in sterilized PBS (pH = 7.2) to a total volume of 300 μL, and then a total of 300 μL of the IRBP emulsion was subcutaneously administered to each healthy female Lewis rat (6–8-week-old) to induce EAU. On day 0, subcutaneous immunization with IRBP emulsion was administered at five sites for each rat in the EAU, PA, and LXD groups, while those in the NC group received 300 µL of sterilized PBS (pH = 7.2) supplemented with only 150 µL of CFA and 100 µg of TB. After EAU induction, rats in the L-LXD group, the M-LXD group, and the H-LXD group were orally administered LXD extract once daily for consecutively 12 days (from day 0 to day 12). At the same time, the rats in the PA group were treated with PA hormone by gavage once daily from day 0 to day 12, while the rats in both NC and EAU groups received orally identical volumes of sterilized PBS once every day.

### 2.10. Clinical and Histopathological Evaluation

On day 12 postimmunization, the inflammatory response in the anterior segment of the eyes was captured with a Genesis-D camera (Kowa Company Ltd., Japan). Further, anesthesia was induced in the rats via intraperitoneal administration of 3% pentobarbital sodium salt (50 mg/kg) until the rats were in a deep coma and the pain reflex disappeared. After fixation with a 4% formaldehyde solution for 24 h, the isolated eyes were subsequently embedded in paraffin wax. Subsequently, the samples were sliced and stained with a H&E solution. Finally, the slice was viewed under a microscope (Ti; Nikon Corporation, Tokyo, Japan). The degree of inflammation was scored according to the criteria on a scale of 0–4 (Supporting Information 1: Table S1) [29].

### 2.11. Cell Processing

The spleen tissue and lymph nodes were harvested from both the NC and EAU rats, and the whole eyeballs were removed using vitrectomy extraction from the rats at day 12 postimmunization under anesthesia by administration of 3% pentobarbital sodium salt (50 mg/kg) through intraperitoneal injection followed by exsanguination of abdominal aorta. First, the cell suspension from each tissue was isolated by grounding, filtered by a stainless 200 mesh cell sieve, and incubated at 37°C for 1 h. Once the cell suspension was filtered through a nylon wool column, ~1 × 10^6^ T lymphocytes were isolated via the employment of the rat lymphocyte separation solution. Then the lymphocytes were seeded in 24-well flat plates (Nest Biotechnology, Wuxi, China), and gently blended with 6 μL of cell activation cocktail, 4 μL of protein transport inhibitor, 2 mL of RPMI 1640 medium (including 100 μL of FBS plus 2 μL of β-mercaptoethanol) with 10 μL of IRBP (10 mg/mL) [30], and 1 μL of recombinant rat IL-2 (10 ng/mL).

### 2.12. Cell Grouping and Transfection In Vitro

Collected T lymphocytes were exposed to various concentrations of LXD-containing serum. Among them, lymphocytes from the NC group and EAU group were treated with 2% control serum (40 μL of serum from the NC rats), while cells from the 0.5% LXD group, 1% LXD group, and 2% LXD group were treated with 0.5%, 1%, and 2% of LXD-containing serum (i.e., 10, 20, and 40 μL of LXD-containing serum), respectively. All lymphocytes in each group were further cultured at 37°C for 48 h for the determination.

To validate whether the LXD formula has an inhibitory effect on Notch signaling activation through upregulating miR-30b-5p, we performed an in vitro cell transfection experiment. The cells of the spleens, lymph nodes, and eye tissues isolated from the NC and EAU rats (after EAU induction for 12 days) were divided into the NC, EAU, miR-30b-5p mimic, miR-30b-5p inhibitor, miR-30b-5p mimic + LXD, miR-30b-5p inhibitor + LXD, mimic control, and inhibitor control groups. According to the manufacturer's protocol, rno-miR-30b-5p mimic (50 nmol) and rno-miR-30b-5p inhibitor (100 nmol, RiboBio, China) were transfected separately into T lymphocytes (5 × 10^5^ cells/well, final volume: 2 mL) from spleen, lymph nodes or eyes with riboFECT CP reagent kit. Cells were further cultured for 48 h. In the miR-30b-5p mimic + LXD and miR-30b-5p inhibitor + LXD groups, 2% LXD-containing serum (40 μL) were separately added when T cells were transfected with either mimic or inhibitor solutions, and all lymphocytes in each group were further cultured at 37°C for 48 h for the determination.

### 2.13. The Assessment of Th1, Th2, Th17, and Treg Cell Frequencies

The collected cells in each group were first rinsed with PBS and then centrifuged at 500 × *g* for 6 min. All lymphocytes were surface stained with FITC-CD4 (Cat.#11-0040-82, LOT: 2,386,305). Intracellular staining was performed using PE-IFN-γ (Cat.#507806, LOTB325446), PE-IL-4 (Cat.#511906, LOTB332499), and PE-IL-17A (Cat.#12-7177-81, LOT1995433) to characterize the frequencies of Th1, Th2, and Th17 cells, respectively. Treg lymphocytes were also surface labeled with APC-CD25 (Cat.#202105, LOT: B207938), and then labeled intracellularly with PE-Foxp3 (Cat.#12-5773-82, LOT: 2430489). The Th1/Th2 and Th17/Treg proportions were analyzed using flow cytometry (BD FACSVerse, USA).

### 2.14. Expression of RORγt, Foxp3, Notch1, DLL4, IL-17A, IL-10 mRNAs, and miR-30b-5p

Reverse mRNA transcription and miRNA first strand cDNA synthesis after extraction of total RNA and miRNA. q-PCR analysis for mRNA and miRNA levels was performed using SYBR qPCR Master Mix with the LightCycler 480 II instrument.  Table 1 displayed primer sequences for target genes. The internal reference utilized for rno-miR-30b-5p was U6, while other target genes were GAPDH. In transfection experiments, the internal reference used for the target genes was β-actin. q-PCR reaction was performed according to the following parameters: 94°C for 5 min for 1 cycle, 94°C for 5 s, 57°C for 15 s, and 72°C for 10 s for 45 cycles. The RORγt, Foxp3, Notch1, DLL4, IL-17A, IL-10 mRNAs, and miR-30b-5p levels were determined using the 2−*ΔΔ*Ct calculation formula after q-PCR detection.

### 2.15. The Measurement of the ROR*γ*t, Foxp3, Notch1, DLL4, IL-17A, IL-10, IFN-γ, and IL-4 Protein Levels

The alteration in proinflammatory cytokines either within the spleen, lymph nodes, and eye tissues were quantified following EAU induction for 12 days (in vivo) or cultured cells treated with different concentrations of LXD-containing serum for 48 h (in vitro), both in vitro and in vivo samples were homogenized in liquid nitrogen and dissolved in 350 μL RIPA lysis buffer. Afterward, the samples underwent sonication on ice for 20 min, followed by centrifugation at 8000 × *g* at 4°C for 20 min. The resulting supernatants were then harvested. The RORγt, Foxp3, Notch1, DLL4, IL-17A, IL-10, IFN-γ, and IL-4 protein levels for each sample were quantified by ELISA using a multifunctional microplate reader.

### 2.16. Statistical Analysis

All experiments repeated thrice. The results were presented as mean ± S.D. (standard deviation) and statistical analysis was performed using SPSS statistical software (SPSS for Windows, version 22.0, IBM-SPSS, Chicago, IL, USA). One-way ANOVA was conducted following LSD multiple comparison tests. Significance was defined as *p* < 0.05.

## 3. Results

### 3.1. Disease Targets

**Figure 1:**
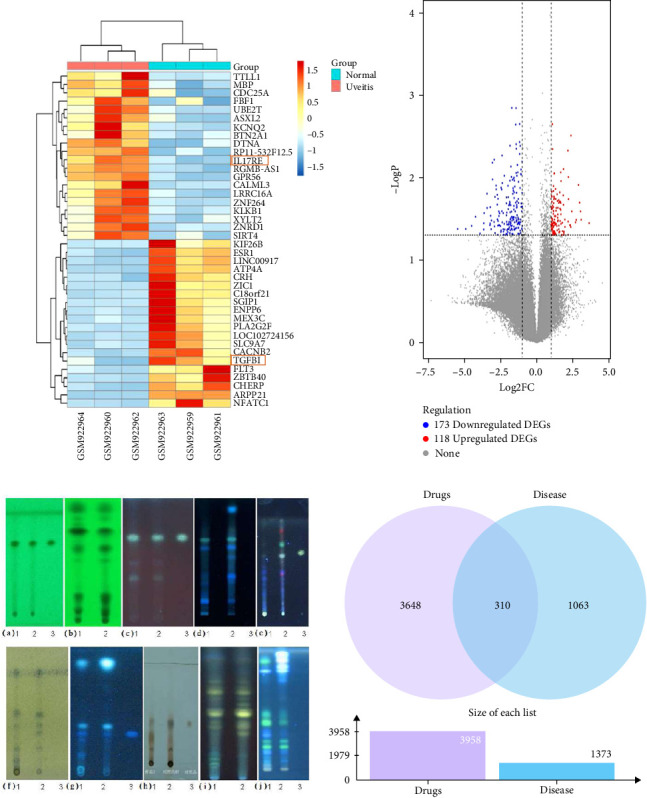
Potential therapeutic targets and bioactive components of LXD. (A) Heat map of the top 20 upregulated DEGs and the top 20 downregulated DEGs. DEGs in GSE37588. GSM922960, GSM922962, and GSM922964 are from uveitis tissues, and GSM922959, GSM922961, and GSM922963 are from normal tissues. Red rectangles indicate upregulated molecules and blue rectangles indicate downregulated molecules. (B) Volcano plot of differentially expressed genes in GSE37588. The red plots represent upregulated genes, the gray plots represent genes with no significant difference, and the blue plots represent downregulated genes. (C) Identification of the active ingredients from LXD boil-free granules characterized by TLC. (a) 1: Boil-free granules of *Gentiana scabra Bunge*; 2: standard reference of *Gentiana scabra Bunge*; 3: standard reference substance of gentiopicroside. (b) 1: Boil-free granules of *Scutellaria baicalensis Georgi*; 2: standard reference of *Scutellaria baicalensis Georgi*. (c) 1: Boil-free granules of *Gardenia jasminoides J.Ellis*; 2: standard reference of fructus gardenia; 3: standard reference substance of geniposide. (d) 1: Boil-free granules of *Alisma plantago-aquatica subsp. orientale* (*Sam*.) *Sam*.; 2: Rhizoma Alismatis; 3: negative control. (e) 1: Boil-free granules of *Akebia trifoliata* (*Thunb*.) *Koidz*.; 2: standard reference of *Akebia trifoliata* (*Thunb*.) *Koidz*.; 3: standard reference substance of oleanolic acid. (f) 1: Boil-free granules of *Plantago asiatica L*.; 2: standard reference of *Plantago asiatica L*.; 3: negative control. (g) 1: Boil-free granules of *Angelica sinensis* (*Oliv*.) *Diels*; 2: standard reference of *Angelica sinensis* (*Oliv*.) *Diels*; 3: standard reference substance of Ferulic acid. (h) 1: Boil-free granules of *Rehmannia glutinosa* (*Gaertn*.) *DC*.; 2: standard reference of *Rehmannia glutinosa* (*Gaertn*.) *DC*.; 3: standard reference substance of Catalpol. (i) 1: Boil-free granule of *Bupleurum chinense DC*.; 2: standard reference of *Bupleurum chinense DC*.; (j) 1: Boil-free granules of *Glycyrrhiza glabra L*.; 2: standard reference of *Glycyrrhiza glabra L*.; 3: negative control. (D) Venn diagram of the crossover target genes between potential therapeutic targets of LXD and uveitis-related genes.

A total of 1697 disease targets were mined using OMIM and GeneCards. In the gene expression profile of GSE37588, a total of 291 DEGs were identified, which is composed of 118 upregulated and 173 downregulated DEGs (Figure 1B). In the gene expression data profile of GSE37588, we picked the top 20 upregulated DEGs and the top 20 downregulated DEGs between uveitis and the normal tissues, which were visualized by heat map (Figure 1A). After the removal of duplicate therapeutic targets, we finally obtained 1373 uveitis-related targets (Figure 1D).

### 3.2. ADME Parameters of Bioactive Components and Potential Therapeutic Targets

TLC had rich information, clear spots, and good separation. In the chromatogram, spots of the same color can be observed in LXD boil-free granules, appearing at positions that correspond to those in reference chromatogram of medicinal material. A specific spot was identified in Radix Gentianae, and its location and color matched those of standard reference substance of gentiopicroside (Figure 1C).

After conducting ADME calculations in TCMSP, a total of 113 potentially bioactive components were identified (Supporting Information 2: Table S2). The average OB value of these compounds was 35.28, with a median of 34.47, and a range of 1.52–79.91. The average DL value of these compounds was 0.41, with a median of 0.27, and a range of 0.02–0.86. These results indicated that the active compounds of LXD can enter the blood smoothly through oral administration for preparation of medicated serum, and LXD was reasonable as a decoction for intake.

After processing a total of 113 bioactive components from LXD through PubChem, Swiss Target Prediction, and Pharmmapper, we obtained 3958 potential candidate therapeutic targets for the bioactive ingredients (Figure 1D).

### 3.3. The Construction of PPI Network Based on Common Targets

In the present study, we obtained 310 crossover genes between the bioactive ingredients and uveitis-related targets (Figure 1D). The original PPIs of drug-disease crossover genes consisted of 308 nodes and 1342 edges, and the average node degree was 8.71 (Supporting Information 3: Figure S1). After the identification of hub genes using three kinds of algorithm in the cytoHubba plugin, 168 genes were acquired, which may play an important role (Supporting Information 4: Figure S2). The network showed that LXD exerted therapeutic effects upon valuable targets participated in the genesis and development of uveitis, including Notch1, IL-6, IL-2, TNF, and IL-10.

### 3.4. Bioinformatics Annotation of the Drug-Disease Crossover Genes and Prediction of Corresponding miRNAs

**Figure 2:**
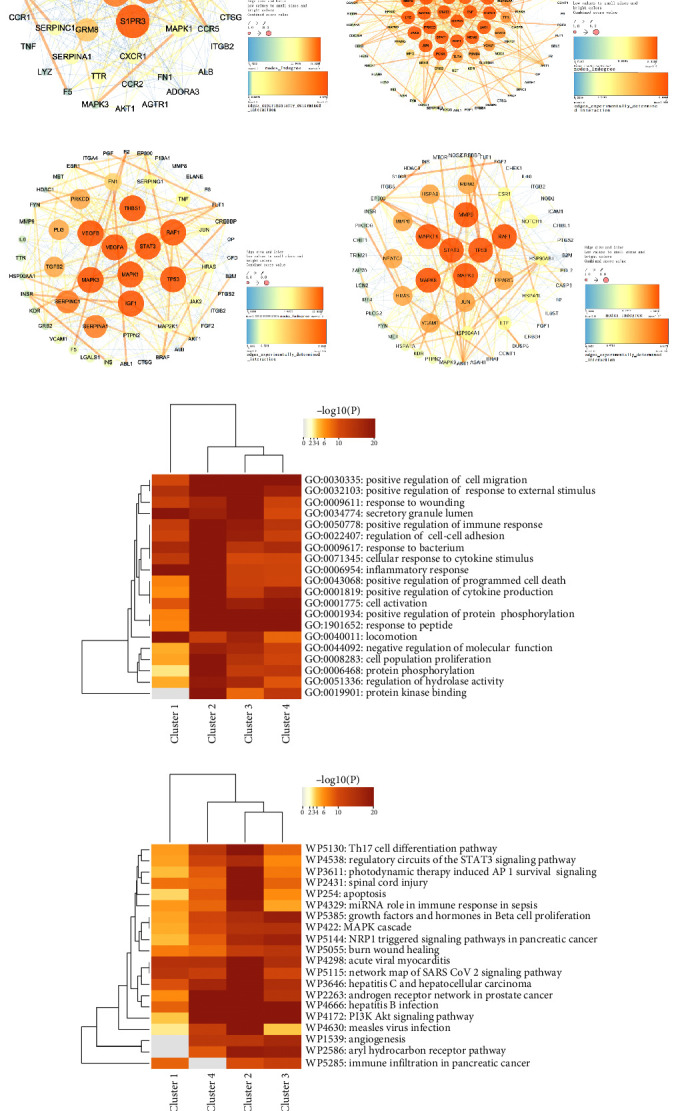
Bioinformatics annotation of the drug-disease crossover genes. (A–D) The highest-scoring top four modules obtained from the PPI network comprising 168 drug-disease crossover hub genes. (E) Heatmaps of the GO-based enrichment terms for genes within the top four modules. (F) Heatmaps of biological pathways (WikiPathway) enrichment for genes within the top four modules. The heatmaps were generated based on Kappa-statistical similarities among their gene memberships. Color key in heatmaps, ranging from gray to brown, represented high to low *p*-values, respectively.

We divided the hub gene network into 10 functional modules by the MCODE plugin to find the clusters most associated with the therapeutic effects of LXD. Supporting Information 5: Table S3 includes the parameters utilized in different modules. We wondered whether these modules exhibited similar functional patterns associated to uveitis, and thus imported genes within the top four modules (Figure 2A–D) with the highest scores as four clusters into Metascape. The imported clusters showed significant overlaps primarily in immune-related GO terms, including regulation of immune response (GO:0050778), cytokine production (GO:0001819), and cell population proliferation (GO:0008283) (Figure 2E). Furthermore, these overlapped biological processes were mainly linked to signaling pathways involving Th17 cell differentiation (WP5130) and the role of miRNA in the immune response (WP4329) (Figure 2F). Cluster 2 (Module 2) (Figure 2B), comprising of 119 nodes and 761 edges with a score of 12.898, represented a higher enrichment significance in bioinformatics annotation and was specifically chosen as the subnetwork module most closely associated with the therapeutic effects of LXD.

**Figure 3:**
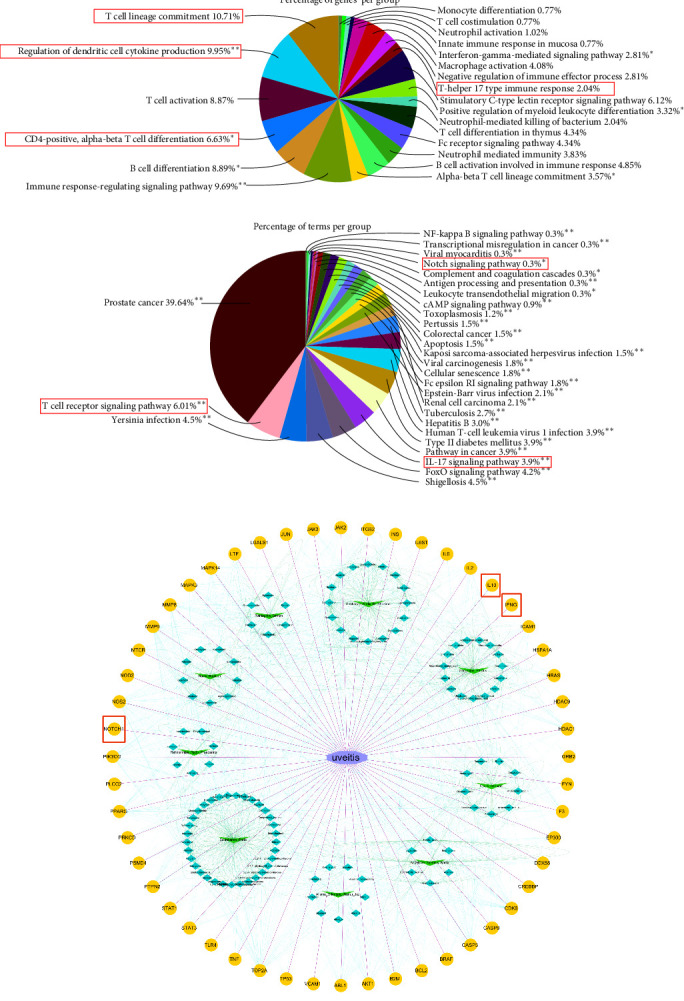
Bioinformatics annotation of the subnetwork module most closely associated with the therapeutic effects of LXD. (A) Pie chart of immune system process terms in the ClueGo. (B) Pie chart of KEGG annotation. (C) Compound-target-disease (C-T-D) network. The green nodes in the V-shaped figure represent herbs, while the green nodes in diamond shape represent their active compounds. The yellow nodes in the ellipse-shaped figure symbolized 52 genes closely related to T-cell lineage commitment that were identified through GO terms enrichment (immune system process). Meanwhile, edges represent the three types of interactions between them.

Based on immune system process terms in the ClueGo plugin, genes in Module 2 (Cluster 2) were related to 113 immunological terms. The terms mainly enriched on T cell-lineage commitment (Figure 3A). The KEGG enrichment analysis revealed that the key subnetwork module was mainly enriched on the T cell receptor signaling pathway, IL-17 signaling pathway, and Notch signaling pathway (Figure 3B). After identifying a function group comprising 52 genes closely involved in the term of T cell-lineage commitment, we obtained a C-T-D network (Figure 3C). Radix Gentianae (*Gentiana scabra* Bunge) and Radix Scutellariae (*Scutellaria baicalensis Georgi*) are drugs that exert major therapeutic effects on the main diseases in accordance with the TCM theory and network pharmacology findings. In the C-T-D network, 1-o-β-d-glucopyranosylamplexin and Gentiatibetine are active ingredients identified from Radix Gentianae. Wogonin, moslosooflavone, and nothosmyrnol are active ingredients isolated and identified from Radix Scutellariae. All of them directly affect Notch1 receptor and act on representative Notch signaling components to reduce the production of proinflammatory mediators, thereby playing a major role in immune regulation. In addition, gentiopicrin (gentiopicroside) isolated from Radix Gentianae, together with sucrose and baicalin, which are active ingredients identified from Radix Scutellariae, are compounds mediating the antiinflammatory IL-10 production.

**Figure 4:**
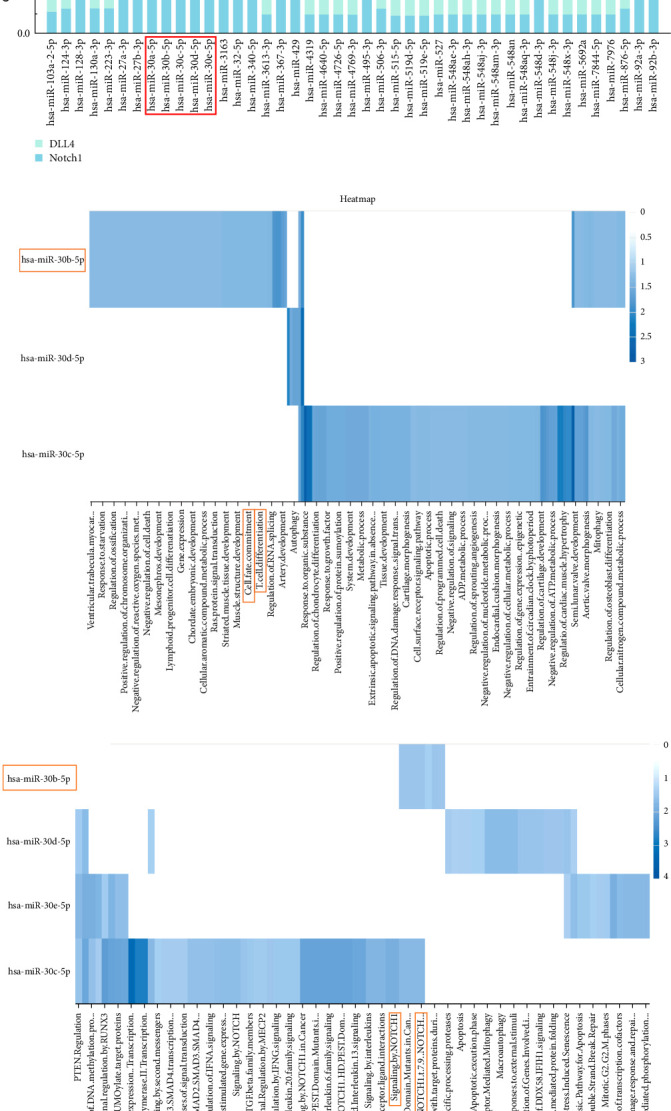
Computational prediction of corresponding miRNAs. (A) The Venn diagram illustrating the shared miRNAs associated Notch1 and DLL4. (B) Confidence score of the miRNA–mRNA interaction of the 43 shared miRNAs associated Notch1 and DLL4. (C) Heat maps of GO annotation (biological processes) of the miR-30 family. (D) Heat maps of reactome pathway annotation of the miR-30 family. (E) Computational prediction of the miR-30b-5p binding sites on each transcript of Notch1 and DLL4.

In order to elucidate the influence of LXD on the inflammatory response of EAU, we conducted a prediction analysis of miRNAs capable of regulating important components of the Notch signaling pathway. This analysis was carried out based on the perspective of miRNA–mRNA interaction, as indicated by the findings presented in Figure 4 regarding the role of miRNA in the immune response (WP4329). By searching the RNAInter database, 194 miRNAs capable of regulating Notch1 and 187 miRNAs capable of regulating DLL4 (Figure 4A) were identified based on the integrative confidence score. In the 43 overlapping miRNAs (Figure 4B), we identified that the miR-30 family as significant miRNAs involved in T-cell lineage commitment and Notch signaling pathway (Figures 4C,D). We found that miR-30b-5p was focused on the enrichment of GO terms related to T-cell lineage commitment (Figure 4C) and Notch signaling pathway (Figure 4D). The results of computational prediction by TargetScan displayed the sites of miR-30b-5p for each transcript of Notch1 and DLL4, and further indicated the miRNA–mRNA interaction between the miR-30 family, Notch1 and DLL4 in the Notch signaling pathway (Figure 4E). In the present study, we selected miR-30b-5p to be validated on their mRNA targets (i.e., Notch1, DLL4) in the following experiment.

### 3.5. The Efficacy of LXD on the Pathogenesis and Inflammation of EAU In Vivo

**Figure 5:**
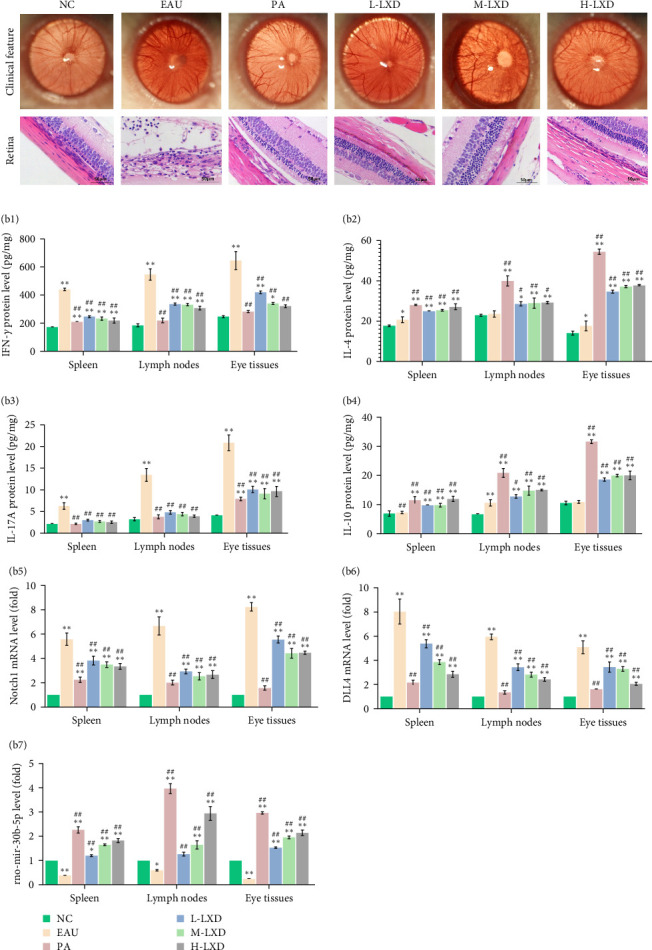
Evaluations of the clinical and pathological features in NC, EAU, PA, L-LXD, M-LXD, and H-LXD groups in vivo on day 12 after immunization. (A) The representative images of intraocular inflammation captured by a Genesis-D camera and H&E staining in the retina of histopathological alterations in the six groups (bar = 50 μm). (B) The expression of the relevant inflammatory cytokines, Notch signaling-related molecules, and miR-30b-5p in the six groups. The relevant inflammatory cytokine levels (IFN-γ [b1], IL-4 [b2], IL-17A [b3], and IL-10 [b4]) were measured by ELISA, and Notch signaling-related molecule mRNA levels (Notch1 [b5], DLL4 [b6]) and miR-30b-5p expression (b7) were determined by q-PCR.

The peak of the ocular inflammatory response in the EAU and LXD groups was observed on the 12th day after immunization. In normal rat eyes, there was no infiltration of inflammatory cells and no dilation or congestion of iris blood vessels observed. The iris vessels of EAU rats were severely congested with cloudy anterior chambers, fibrin exudation, obscured pupil, and ocular protrusion compared to NC subjects (Figure 5A). Histopathological examination showed full-thickness retinal destruction with inflammatory cells infiltration, while rats in the LXD groups appeared weaker inflammation, which displayed mild dilatation of the iris vasculature, reduced fibrin exudation in the anterior chamber, and visible pupils (Figure 5A).

The levels of IFN-γ (Figure 5B b1) and IL-17A (Figure 5B b3) from the spleen, lymph nodes, and eye tissues in the NC, EAU, and LXD groups showed similar expression patterns related to immune reactions. The EAU group exhibited markedly elevated trends in the protein expression levels of IFN-γ and IL-17A in the EAU group and showed significantly increased trends on day 12 postimmunization as compared to the NC group (all *p* < 0.01). The IFN-γ and IL-17A protein levels remained elevated in the LXD groups compared to the NC group, but reduced compared to the EAU group (all *p* < 0.01). Despite the elevation of IL-4 (Figure 5B b2) and IL-10 (Figure 5B b4) in the EAU group, LXD administration could further intensify the IL-4 and IL-10 expression (all *p* < 0.05), suggesting the recovery of EAU rats.

Notch1 (Figure 5B b5) and DLL4 (Figure 5B b6) expression was increased in the spleen, lymph nodes, and eye tissues of EAU rats (all *p* < 0.01), while it decreased in rats treated with different doses of LXD (all *p* < 0.01). The miR-30b-5p (Figure 5B b7) expression declined in EAU rats (all *p* < 0.05), and LXD administration with various doses effectively enhanced miR-30b-5p expression compared to the EAU group (all *p* < 0.01).

### 3.6. Changes in the Expression of ROR*γ*t, Foxp3, Notch1, DLL4, IL-17A, and IL-10 mRNAs, as Well as miR-30b-5p After Intervention With Various Concentrations of LXD-Containing Serum

**Figure 6:**
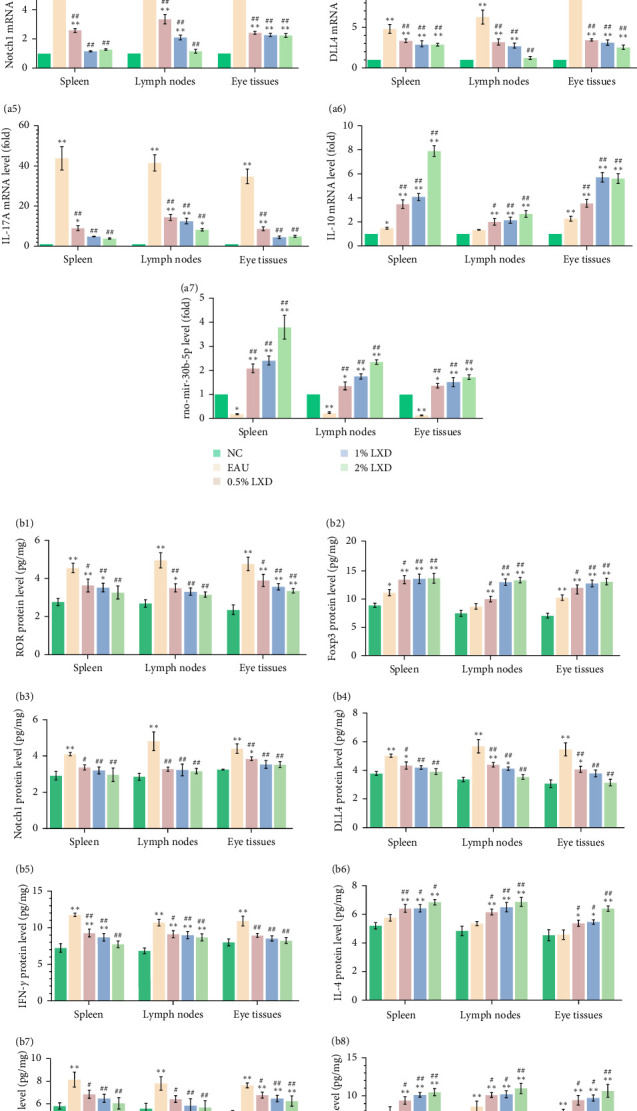
Expression of miR-30b-5p and Notch signaling-related molecules and the relevant inflammatory cytokines in the NC, EAU, 0.5% LXD, 1% LXD, and 2% LXD groups after different treatments in vitro for 48 h. The cells were cultured with exposure to various concentrations of LXD-containing serum (i.e., 0, 10, 20, and 40 μL/mL) for 48 h. (A) RORγt (a1), Foxp3 (a2), Notch1 (a3), DLL4 (a4), IL-17A (a5), IL-10 (a6) mRNA levels and miR-30b-5p (a7) expression determined by q-PCR. (B) RORγt (b1), Foxp3 (b2), Notch1 (b3), DLL4 (b4), IFN-γ (b5), IL-4 (b6), IL-17A (b7), and IL-10 (b8) protein levels determined by ELISA. Data were presented as mean ± SD. *⁣*^*∗*^*p* < 0.05 and *⁣*^*∗∗*^*p* < 0.01 compared with the NC group; ^#^*p*<0.05 and ^##^*p* < 0.01 compared with the EAU group. One-way ANOVA was used to analyze the data and post hoc analysis was performed using the LSD method.

The miR-30b-5p significantly declined in the EAU group (all *p* < 0.05, Figure 6A a7) relative to the NC group. However, it was elevated following the administration of 0.5% LXD, 1% LXD, and 2% LXD treatments compared to the expression level observed in the EAU group (all *p* < 0.01).

The EAU group showed increased expression levels of RORγt, Foxp3, Notch1, DLL4, IL-17A, and IL-10 (Figure 6A a1–a6) mRNAs in contrast with the NC group. In addition, the expression levels of RORγt, Notch1, DLL4, and IL-17A mRNAs were reduced in the 0.5%, 1%, and 2% LXD groups compared to those in the EAU group (all *p* < 0.01). By contrast, LXD-treated groups showed a significant increase in Foxp3 (Figure 6A a2) and IL-10 (Figure 6A a6) mRNA levels compared to the EAU group (all *p* < 0.05).

### 3.7. Alterations in the ROR*γ*t, Foxp3, Notch1, DLL4, IL-10, IL-17A, IFN-*γ*, and IL-4 Protein Levels After Intervention With Various Concentrations of LXD-Containing Serum In Vitro

In the intracellular and culture supernatants, the expression of a variety of proteins including RORγt (Figure 6B b1), Foxp3 (Figure 6B b2), Notch1 (Figure 6B b3), DLL4 (Figure 6B b4), IL-17A (Figure 6B b7), IL-10 (Figure 6B b8), IFN-γ (Figure 6B b5), and IL-4 (Figure 6B b6) in the EAU group exhibited a notable increase compared to the NC group. By contrast, after treatment with 0.5% LXD, 1% LXD, and 2% LXD for 48 h, the protein levels of RORγt, Notch1, DLL4, IL-17A, and IFN-γ were markedly decreased (all *p* < 0.05), whereas the expression levels of Foxp3, IL-10, and IL-4 were considerably elevated (all *p* < 0.05).

### 3.8. Changes in Th1, Th2, Th17, and Treg Frequencies After Intervention With Various Concentrations of LXD-Containing Serum In Vitro

**Figure 7:**
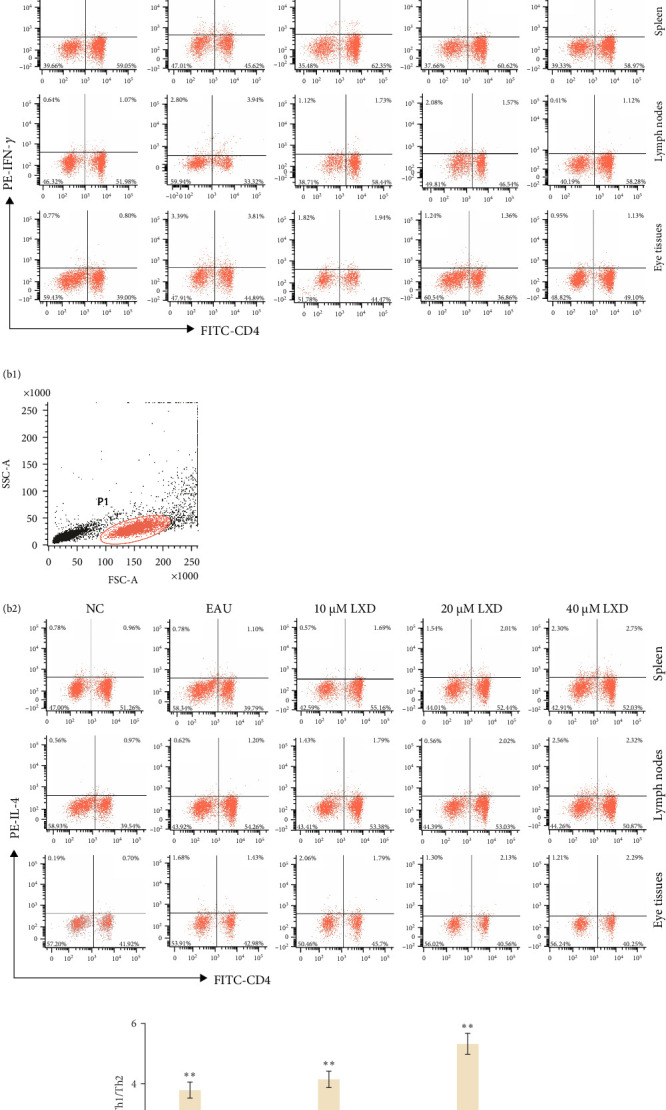
The changes of Th1 and Th2 levels in the NC, EAU, 0.5% LXD, 1% LXD, and 2% LXD groups after different treatments in vitro for 48 h. After different treatments for 48 h, the samples were stained with relevant fluorescent antibodies and were detected by flow cytometry. (A) T lymphocytes (a1), and representative scatter-grams of the frequencies of IFN-γ-producing CD4^+^ T cells (a2). T lymphocytes were gated in P1 of (a1), (a2) was from P1, and CD4^+^ IFN-γ^+^ T cells (Th1 cells) were shown in the right quadrant of (a2). (B) T lymphocytes (b1), and representative scatter-grams of the frequencies of IL-4-producing CD4^+^ T cells (b2). T lymphocytes were gated in P1 of (b1), (b2) was from P1, and CD4^+^ IL- 4^+^ T cells (Th2 cells) were shown in the right quadrant of (b2). (C) The histogram analysis of Th1/Th2 ratios in the spleen, lymph nodes, and eyes after different treatments for 48 h was presented as mean ± SD. *⁣*^*∗*^*p* < 0.05 and *⁣*^*∗∗*^*p* < 0.01 compared with the NC group; ^#^*p* < 0.05 and ^##^*p* < 0.01 compared with the EAU group. One-way ANOVA was used to analyze the data and post hoc analysis was performed using the LSD method.

**Figure 8:**
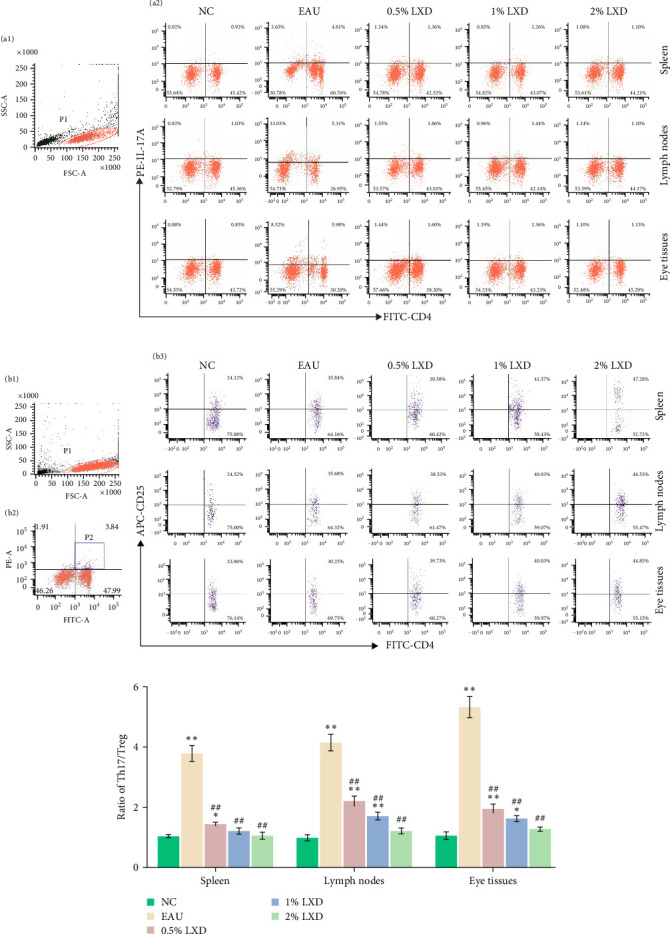
The changes of Th17, Treg levels in NC, EAU, 0.5% LXD, 1% LXD, and 2% LXD groups after different treatments in vitro for 48 h. After different treatments for 48 h, the samples were stained with relevant fluorescent antibodies and were detected by flow cytometry. (A) T lymphocytes (a1), and representative scatter-grams of the frequencies of IL-17A-producing CD4^+^ T cells (a2). T lymphocytes were gated in P1 of (a1), (a2) was from P1, and CD4^+^ IL-17A ^+^T cells (Th17 cells) were shown in the right quadrant of (a2). (B) Treg levels. T lymphocytes (b1), CD4^+^ T lymphocytes (b2), and representative scatter-grams of the expression of CD25 on CD4^+^ Foxp3^+^ T cells (b3). T lymphocytes were gated in P1 of (b1), (b2) was from P1, CD4^+^ Foxp3^+^T cells were gated in P2 of (b2), and CD4^+^CD25^+^Foxp3^+^T cells (Treg cells) were shown in the right quadrant of (b3). (C) The histogram analysis of Th17/Treg ratios in the spleen, lymph nodes, and eyes after different treatments for 48 h was presented as mean ± SD. *⁣*^*∗*^*p* < 0.05 and *⁣*^*∗∗*^*p* < 0.01 compared with the NC group; ^#^*p* < 0.05 and ^##^*p* < 0.01 compared with the EAU group. One-way ANOVA was used to analyze the data and post hoc analysis was performed using the LSD method.

Flow cytometry outcomes demonstrated the Th1, Th2, Th17, and Treg frequencies in the EAU group were significantly higher than those in NC group. We noted that compared with the EAU group, the Th1 subsets (Figure 7A) and Th17 subsets (Figure 8A) in 0.5%, 1%, and 2% drug-containing serum groups decreased, whereas Th2 subsets (Figure 7B) and Treg cell subsets (Figure 8B) increased. Th1/Th2 (Figure 7C) and Th17/Treg ratios (Figure 8C) showed significant reduction (*p* < 0.01) with LXD intervention compared to the EAU group. Nevertheless, the ratios of Th1/Th2 and Th17/Treg were still higher than those in the NC group and tended to restore to normal balance. It was noted that LXD treatment could efficiently reregulate the plasticity between Th1/Th2 and Th17/Treg subsets to further restore their balance, and finally improve immune homeostasis.

### 3.9. Expression of Notch Signaling-Related Molecules and the Corresponding Inflammatory Cytokines After Cell Transfection In Vitro

**Figure 9:**
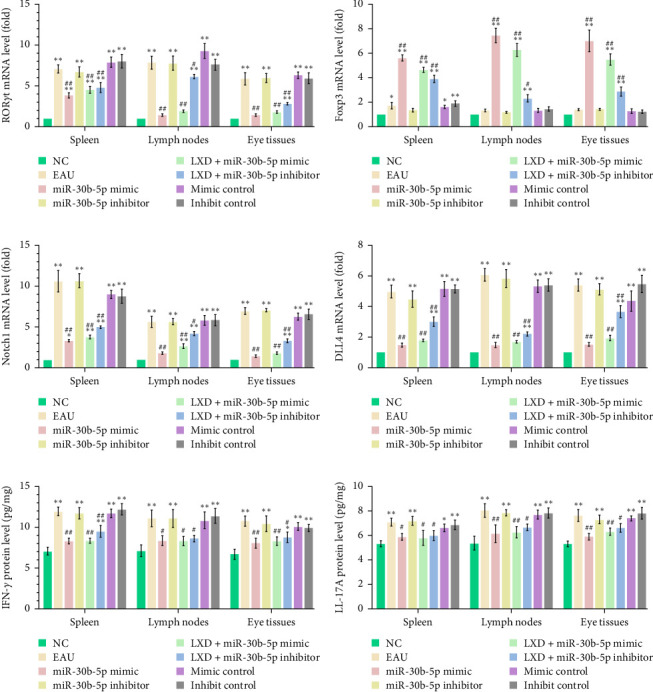
The expression of Notch signaling-related molecules and the relevant inflammatory cytokines after treatment in vitro with rno-miR-30b-5p mimic, inhibitor, rno-miR-30b-5p mimic accompanied by LXD, rno-miR-30b-5p inhibitor accompanied by LXD, mimic negative, and inhibitor negative to T cells. (A) RORγt, (B) Foxp3, (C) Notch1, (D) DLL4 expression determined by Q-PCR. (E) IFN-γ and (F) IL-17A protein levels determined by ELISA. Data were presented as mean ± SD. *⁣*^*∗*^*p* < 0.05 and *⁣*^*∗∗*^*p* < 0.01 compared with the NC group; ^#^*p* < 0.05 and ^##^*p* < 0.01 compared with the EAU group. One-way ANOVA was used to analyze the data and post hoc analysis was performed using the LSD method.

The groups treated with miR-30b-5p mimic and miR-30b-5p mimic + LXD exhibited a significant decrease (all *p* < 0.01) in the expression of the genes RORγt (Figure 9A), Notch1 (Figure 9C), and DLL4 (Figure 9D), as well as the proteins IFN-γ (Figure 9E) and IL-17A (Figure 9F), in comparison to the EAU groups. However, the gene level of Foxp3 (Figure 9B) was increased (*p* < 0.01).

The gene levels of RORγt, Foxp3, Notch1, and DLL4 (Figures 9) in the miR-30b-5p inhibitor group increased compared to those in the NC group. The coadministration of miR-30b-5p inhibitor and LXD induced a significant decrease in RORγt (Figure 9A), Notch1 (Figure 9C), and DLL4 (Figure 9D) levels (all *p* < 0.05), as well as an increase in Foxp3 level (Figure 9B) (*p* < 0.05). Elevated levels of inflammatory cytokines were found to be significantly higher in the miR-30b-5p inhibitor-treated group when compared to the NC group (all *p* < 0.01). However, no significant difference was found in comparison with the EAU group (all *p* < 0.05). The expression of IFN-γ and IL-17A proteins in the spleen, lymph node, and eye tissues showed a notable decrease (all *p* < 0.05) in the miR-30b-5p inhibitor + LXD group compared to the EAU group (all *p* < 0.05). These results showed that LXD decreased the expression of Notch signaling-associated molecules through upregulating miR-30b-5p, and thus inhibited the activation of the Notch signaling pathway.

### 3.10. Th1, Th2, Th17, and Treg Frequencies After In Vitro Cell Transfection

**Figure 10:**
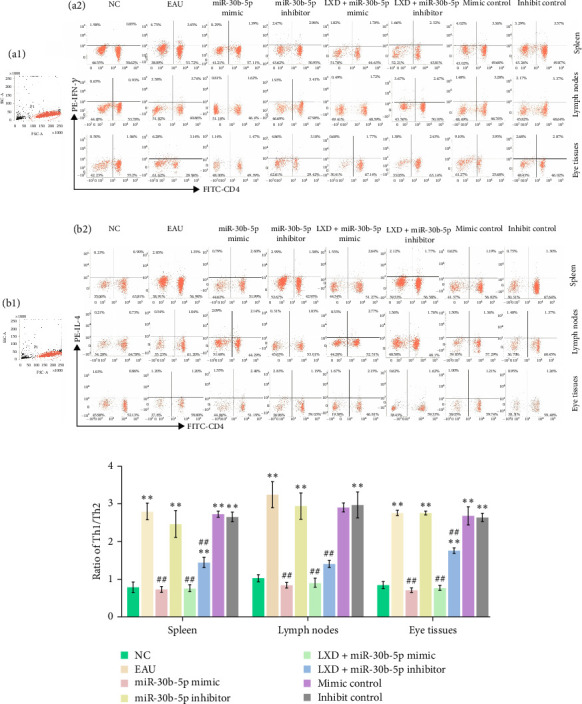
The changes of Th1, Th2 levels in the NC, EAU, rno-miR-30b-5p mimic, rno-miR-30b-5p inhibitor, rno-miR-30b-5p mimic + LXD, rno-miR-30b-5p inhibitor + LXD, mimic control, and inhibitor control groups after different treatment in vitro for 48 h. After different treatments for 48 h, the samples were stained with relevant fluorescent antibodies and were detected by flow cytometry. (A) T lymphocytes (a1), and representative scatter-grams of the frequencies of IFN-γ-producing CD4^+^ T cells (a2). T lymphocytes were gated in P1 of (a1), (a2) was from P1, and CD4^+^ IFN-γ^+^ T cells (Th1 cells) were shown in the right quadrant of (a2). (B) T lymphocytes (b1), and representative scatter-grams of the frequencies of IL-4-producing CD4^+^ T cells (b2). T lymphocytes were gated in P1 of (b1), (b2) was from P1, and CD4^+^ IL- 4^+^ T cells (Th2 cells) were shown in the right quadrant of (b2). (C) The histogram analysis of Th1/Th2 ratios in the NC, EAU, rno-miR-30b-5p mimic, rno-miR-30b-5p inhibitor, rno-miR-30b-5p mimic + LXD, rno-miR-30b-5p inhibitor + LXD, mimic control, and inhibitor control groups after different treatments for 48 h was presented as mean ± SD. *⁣*^*∗*^*p* < 0.05 and *⁣*^*∗∗*^*p* < 0.01 compared with the NC group; ^#^*p* < 0.05 and ^##^*p* < 0.01 compared with the EAU group. One-way ANOVA was used to analyze the data and post hoc analysis was performed using the LSD method.

**Figure 11:**
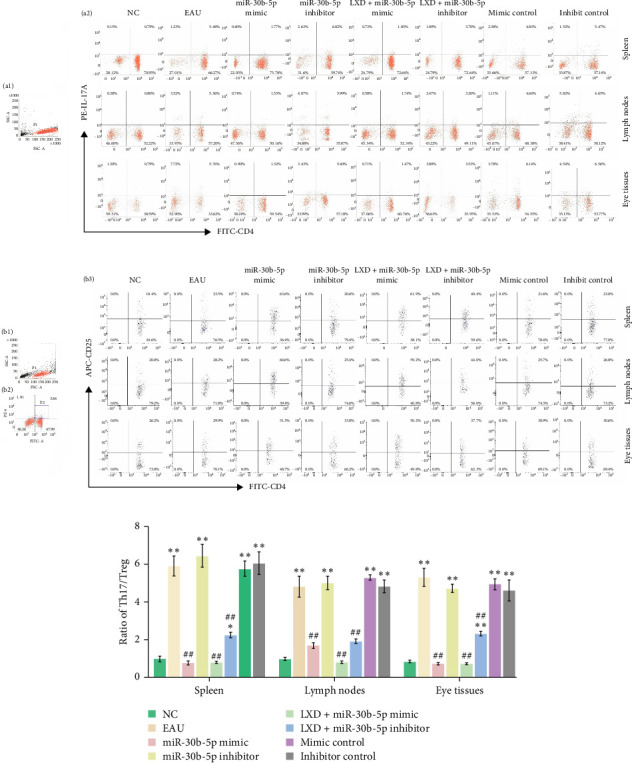
Changes of Th17, Treg levels in the NC, EAU, rno-miR-30b-5p mimic, rno-miR-30b-5p inhibitor, rno-miR-30b-5p mimic + LXD, rno-miR-30b-5p inhibitor + LXD, mimic control, and inhibitor control groups after different treatments in vitro for 48 h. After different treatments for 48 h, the samples were stained with relevant fluorescent antibodies and were detected by flow cytometry. (A) Th17 levels. T Lymphocytes (a1) and representative scatter-grams of the frequencies of IL-17A-producing CD4^+^ T cells (a2). T lymphocytes were gated in P1 of (a1), (a2) was from P1, and CD4^+^ IL-17A^+^ T cells (Th17 cells) were shown in the right quadrant of (a2). (B) Treg levels. T Lymphocytes (b1), CD4^+^T lymphocytes (b2), and representative scatter-grams of the expression of CD25 on CD4^+^ Foxp3^+^ T cells (b3). T lymphocytes were gated in P1 of (b1), (b2) was from P1, CD4^+^ Foxp3^+^ T cells were gated in P2 of (b2), and CD4^+^ CD25^+^ Foxp3^+^ T cells (Treg cells) were shown in the right quadrant of (b3). (C) The histogram analysis of Th17/Treg ratios in the NC, EAU, rno-miR-30b-5p mimic, rno-miR-30b-5p inhibitor, rno-miR-30b-5p mimic + LXD, rno-miR-30b-5p inhibitor + LXD, mimic control, and inhibitor control groups after different treatments for 48 h was presented as mean ± SD *⁣*^*∗*^*p* < 0.05 and *⁣*^*∗∗*^*p* < 0.01 compared with the NC group; ^#^*p* < 0.05 and ^##^*p* < 0.01 compared with the EAU group. One-way ANOVA was used to analyze the data and post hoc analysis was performed using the LSD method.

The ratios of Th1/Th2 (Figure 10C) and Th17/Treg (Figure 11C) displayed a notable decrease in miR-30b-5p mimic and miR-30b-5p mimic + LXD groups as opposed to the EAU group (all *p* < 0.01) and were marginally less than those in the NC group (all *p* < 0.05).

In the miR-30b-5p inhibitor group, the ratios of Th1/Th2 and the ratios of Th17/Treg showed a significant increase compared to the NC group (all *p* < 0.01). Nevertheless, the Th1 (Figure 10A) and Th17 (Figure 11A) cell lineage frequencies exhibited a reduction following a treatment with both miR-30b-5p inhibitor and LXD administration, and the Th2 (Figure 10B) and Treg (Figure 11B) cell frequencies were promoted compared with those of EAU. Nevertheless, the ratios of Th1/Th2 and Th17/Treg were still higher than those in the NC group.

## 4. Discussion

Uveitis is an intraocular autoimmune condition of the iris, ciliary body, and choroid [31]. Classic uveitis treatments (steroids, immunosuppressants, and biologics) have limitations including nonspecific, side effects, and short-term. Drug withdrawal can cause disease relapse and vision loss [4]. It is essential to investigate the immune causes of uveitis and identify safe long-term immunosuppressive treatments. In this context, we employed a comprehensive methodology that includes network pharmacology analysis, bioinformatics analysis, and experimental verification to gain insight into the underlying molecular mechanisms through which LXD modulates uveitis at the miRNA–mRNA interface. Notably, our network pharmacology analysis indicated that T-cell lineage commitment and the Notch signaling pathway are critical in the inflammatory processes associated with uveitis treatment via LXD. Further predictions revealed an interaction between miR-30b-5p and mRNAs related to Notch1 and DLL4.

Based on these findings from network pharmacology and bioinformatics analyses, we then discovered that elevated levels of inflammatory cytokines and Notch signaling-related molecules, along with decreased expression of miR-30b-5p, were identified in the EAU rat model and uveitogenic T lymphocytes. Additionally, we observed dysregulation in the Th1/Th2 and Th17/Treg ratios. Inhibition of miR-30b-5p led to Notch signaling activation and elevated levels of inflammatory cytokines, along with a simultaneous increase in the Th1/Th2 and Th17/Treg ratios. Conversely, increased levels of miR-30b-5p attenuated the ratios of Th1/Th2 and Th17/Treg. LXD effectively downregulated proinflammatory cytokines and upregulated antiinflammatory cytokines in EAU models, correlating with increased miR-30b-5p levels and suppressed Notch signaling activation. Furthermore, LXD restored the balance of Th1/Th2 and Th17/Treg subsets, thereby contributing to immune homeostasis and mitigating ocular inflammation.

It has become increasingly clear that the severity of the inflammatory response is largely affected by the notable increase in neutrophil and macrophage infiltration, as well as the high levels of cytokines, particularly IL-1β, IFN-γ, and TNF in both human and animal models [32]. Macrophages recruit CD4^+^ T cells, and its polarization switch may be reciprocal causation with T cell differentiation, which is extremely susceptible to alterations in cytokine levels within the microenvironment [33]. Oxidative stress and the production of intracellular ROS were shown to be crucial modulators in the process of inflammasome activation [34], and also upregulate the expression of inflammatory chemokines [35].

The activation of Th1 cells is at the core of the EAU, while the activation of Th17 cells is also an important pathogenic event in the development of human inflammatory conditions by secreting IL-17 [36]. In naïve CD4^+^ T cells, Th17, and Treg subsets interrelate with each other in differentiation for their plasticity, and their functions have mutual antagonism [37]. T-cell development accompanied by sequential transcriptional and epigenetic changes leads to T-cell lineage commitment [38]. Foxp3 characterize Treg cells, regulating their plasticity and stability [39]. It is known that T-bet and GATA3 are identified as Th1- and Th2-polarizing transcription factors, respectively, and there is a apparent lineage cross-regulation effect between them. Th2 cells were defined as IL-4 producers, and IL-4 is capable of inducing the expression of GATA3, which is indispensable for Th2 cell differentiation. It also exerts a repressive effect on other lineage-specific transcription factors like T-bet and RORγt [40]. Th17 cells can co-opt T-bet or GATA3 expression to shift phenotype towards Th1 or Th2 cells and acquire characteristics of proinflammatory Th lineages or immuno-regulatory cells [41]. However, by secreting IL-2 and TGF-β, these different Th lineages drive differentiation into suppressive Treg cells, which suppress Th17 differentiation and IL-17 expression to maintain immune homeostasis with secreting IL-10 and TGF-β [42]. Importantly, as specific transcription factors of Th17 and Treg cells, there was an antagonistic interaction between RORγt and Foxp3 [43]. A shift in cytokine expression profiles toward IL-10 can press ocular inflammation in EAU rats [44].

Notch signaling is an important driver in triggering T cell differentiation, committing lymphoid precursors to the T cell fate, and influencing subsequent phases of T cell development [45]. Notch ligand DLL4 is one of the most crucial environmental signals for T cell differentiation. The interaction between DLL4 and Notch1 is critically required to induce cells to shift towards a T-cell-specific gene expression program [46]. Our previous study showed the activation of the Notch signaling pathway participated in the regulation of the plasticity between Th17 and Treg cell lineages in EAU rats [47]. The downregulation of Notch signaling through the use of DAPT resulted in decreased levels of Notch1, DLL4, RORγt, and IL-17, along with the suppression of RORγt transcription. Consequently, the level of Th17 cells were reduced, leading to the restoration of the balance between CD4^+^/CD8^+^ cells and the Th17/Treg ratio [48]. PA treatment significantly reduced ocular and peripheral inflammatory responses and restores Th1/Th2 and Th17/Treg immune homeostasis by orchestrating the Notch signaling pathway [49]. These recent reports, together with our analysis of bioinformatics enrichment, provide evidence that inflammatory cascades mediated by cellular crosstalk between Th subsets and Notch signaling activation are noteworthy contributors to the pathogenesis progress in the occurrence of autoimmune disorders and uveitis. Insights into the antiinflammatory properties of PA treatment have provided substantial implications for designing new classes of antiinflammatory drugs. Notch signaling can potentially serve as a therapeutic target through its regulatory impact on the differentiation of Th subsets in uveitis.

miRNAs are crucial in regulating immunomodulation functions through negative feedback mechanisms [50]. miR-30b-5p acts as a mediator and emphasizes deficiencies in cell migration by targeting critical genes in cell cytoskeleton homeostasis [51]. The inhibition of miR-30b-5p resulted in the activation of Notch1 signaling, and led to an increase in proinflammatory cytokines such as TNF-α and CCL2, as well as the polarization of macrophages towards the M1 phenotype [52]. The increasing miR-30b-5p expression and decreasing DLL4 expression can impede the polarization of inflammatory Th1 cells induced by DLL4-Notch signaling [53]. According to Figure 4, miR-30b-5p possesses various biological functions and acts on multiple immune signaling pathways, leading to the alleviation of inflammatory responses. Additionally, our previous dual luciferase report gene expression assay showed that rno-miR-30b-5p specifically target Notch1 and DLL4 [54], so miR-30b-5p could serve as a promising immunoregulatory approach for the treatment of uveitis.

LXD, a well-known formula composed of 10 Chinese herbal ingredients, is primarily used as an adjunctive therapy for autoimmune diseases. Network pharmacology approache constructs a molecular network mapping the interactions between LXD's multiple complex components and their protein/gene targets, thus comprehensively interpreting the relationship between its targets and therapeutic effects. One meta-analysis demonstrated that LXD yielded a superior cure rate and total effective rate compared to pure Western medicine in eczema treatment. Furthermore, LXD has been shown to reduce disease reactivation and diminish levels of key proinflammatory cytokines, including IL-6, IL-8, and TNF-α, highlighting its immunomodulatory potential [55].

Despite its demonstrated efficacy in modulating immune responses and inflammation, concerns regarding potential long-term side effects, particularly nephrotoxicity, have been associated with LXD. In recent years, reports have emerged linking LXD nephrotoxicity primarily to the historical substitution of *Aristolochia manshuriensis* for the traditional *Akebia trifoliata* (*Thunb*.) *Koidz* in the formula, coupled with instances of misuse (excessive dosage/duration) of *Aristolochia manshuriensis*. Animal studies provide clear evidence: LXD containing a low dose of *Aristolochia manshuriensis* administered to SD rats showed no significant nephrotoxicity over short periods. Conversely, both LXD containing a high dose of *Aristolochia manshuriensis* and single-herb *Aristolochia manshuriensis* significantly elevated serum creatinine and blood urea nitrogen levels after 4 weeks of administration at least. Importantly, the doses of *Aristolochia manshuriensis* employed in experimental studies demonstrating toxicity frequently exceeded the clinically safe dosage limits stipulated in the Pharmacopeia of the People's Republic of China. Therefore, LXD, when formulated correctly using traditional *Akebia trifoliata* (*Thunb*.) *Koidz* or strictly controlled *Aristolochia manshuriensis* within pharmacopeial limits and administered for appropriate durations under TCM guidance, is considered relatively safe [56, 57]. Notably, LXD containing the same dose of *Aristolochia manshuriensis* induced significantly less nephrotoxicity than the equivalent dose of the single herb [58]. This phenomenon is attributed to the fundamental principle of TCM formulas: they consist of multiple herbs with complex components interacting synergistically under the guidance of TCM theory. Modern methodologies like network pharmacology facilitate the dissection of these complex herb–herb interactions, and reveal that evaluating the toxicity of the entire LXD formula based solely on a single component or toxic herb is inconsistent with the long-term practical experience and holistic effects of TCM prescriptions.

In compliance with TCM theory, network pharmacology analyses identify *Gentiana scabra Bunge* and *Scutellaria baicalensis Georgi* as the primary therapeutic components driving LXD's core efficacy against autoimmune conditions. Critically, *Akebia trifoliata* (*Thunb*.) *Koidz/Aristolochia manshuriensis*, the herb implicated in nephrotoxicity, is not a drug that have major therapeutic effects on the main disease or syndrome within LXD. In the TLC chromatogram, gentiopicroside is a significant active compound that has been extracted from *Gentiana scabra Bunge*. It also serves as a valuable quality control parameter for evaluating the quality of *Gentiana scabra Bunge*. Gentiopicroside demonstrated easy absorption in both mice and rats, while both gentiopicroside and baicalin are rapidly absorbed, leading to detectable levels in rat plasma after the oral administration of LXD [59]. Gentiopicroside can be converted to gentianine (gentiatibetine) under the action of human intestinal bacteria [60] and are associated with the downregulation of IL-1β, TNF-α, and IL-6 levels [61, 62]. According to the established C-T-D network, gentianine can affect the Notch1-receptor. Gentiopicrin (gentiopicroside), together with baicalin are the compounds mediating the antiinflammatory IL-10 production. Our previous study has also confirmed that LXD efficiently alleviated the manifestation of EAU [63], and studies in vivo indicated that LXD could substantially suppress the activation of the Notch signaling pathway, leading to the restoration of the imbalanced CD4^+^/CD8^+^ and Th17/Treg cells in EAU within 12 days [64]. Building upon the established efficacy and safety profile of LXD's core components, this study aimed to determine the involvement of miR-30b-5p in the protective effect of LXD against EAU, and to confirm that LXD can induce a significant inhibitory effect on the activation of Notch signaling by upregulating miR-30b-5p expression.

Th1/Th2 and Th17/Treg ratio within T lymphocytes were markedly elevated in the EAU group compared with that of the NC group, accompanied with the activated Notch signaling and downregulated miR-30b-5p level. Th1 and Th17 frequencies significantly decreased following intervention with various concentrations of LXD-containing serum. LXD can decrease the expression of proinflammatory cytokines (Th1 and Th17 frequencies) and increase levels of anti-inflammatory cytokines (Th2 and Treg frequencies), resulting in significant inhibition of pathogenic T cell differentiation.

In the miR-30b-5p mimic and miR-30b-5p mimic + LXD groups, the gene levels of Notch signaling-associated molecules (RORγt, Foxp3, Notch1, and DLL4) and inflammatory cytokines (IL-17A and IFN-γ) expression were reduced. The enhanced expression of Foxp3 and decreased ratios of Th1/Th2 and Th17/Treg also suggested a potential modulation of the immune response. LXD intervention is positively associated with the upregulation of miR-30b-5p, which in turn exerted a regulatory effect on immune cells and the expression of antiinflammatory factors by inhibiting Notch signaling-related factors. Inhibition of miR-30b-5p led to enhanced expression of Notch signaling-associated molecules and inflammatory cytokines. There was a concomitant rise in Th1/Th2 and Th17/Treg ratios. However, when the miR-30b-5p inhibitor was transfected in the presence of LXD administration, it led to a decrease in mRNA levels of Notch signaling-associated molecules (RORγt, Foxp3, Notch1, and DLL4). Additionally, there was a reduction in the subsets of Th1 and Th17 cells. Conversely, this treatment promoted the production of Foxp3 mRNA, resulting in the differentiation of immune cells into Th2 and Treg cell subsets, suggesting that LXD could inactivate Notch signaling by promoting miR-30b-5p generation. LXD could also demonstrate a noteworthy ability to promote the Treg and Th2 cell expansion and restore the imbalanced ratios of Th1/Th2 and Th17/Treg, leading to the suppression of pathogenic T cells differentiation and amelioration of uveitis consequently.

## 5. Conclusions

The TCM LXD can ameliorate EAU by inhibiting Notch signaling activation to regulate Th differentiation and restore the Th1/Th2 and Th17/Treg balance through upregulating miR-30b-5p. Our investigations shifted the focus from network pharmacology to analysis carried out on the perspective of miRNA–mRNA interaction, and combine network pharmacology approach with molecular biology experiments for further verification. These investigations allow us to better understand the underlying molecular mechanisms of LXD treating uveitis.

## Figures and Tables

**Table 1:** Primer sequence of genes.

Gene	Primer sequence	
GAPDH	F:	5′-GACCACAGTCCATGACATCACT-3′
	R:	5′-TCCACCACCCTGTTGCTGTAG-3′

Notch1	F:	5′-ATGGCCCCACCTGCAGACAAGATG-3′
	R:	5′-GGCACGGCAGGCACAGCGATAG-3′

DLL4	F:	5′-CAAGAATAGCGGCAGTGGTCGTAA-3′
	R:	5′-GTAGCGCAGTCTTGTGAGGGTGTT-3′

IL-10	F:	5′-TTCCATCCGGGGTGACAATAA-3′
	R:	5′-TTCTGGGCCATGGTTCTCTGC-3′

IL-17A	F:	5′-TTGCTGCTACTGAACCTGGAG-3′
	R:	5′-GCATGGCGGACAATAGAG-3′

Foxp3	F:	5′- TATGCGGCCCCCTTTCACCTATG-3′
	R:	5′- GGGGCGTTGGCTCCTCTTCTTG-3′

miR-30b-5p	RT:	5′-GTCGTATCCAGTGCGTGTCGTGGAGT CGGCAATTGCACTGGATACGACAGCTGA-3′
	GSP:	5′-GGGCTGTAAACATCCTACAC-3′
	R:	5′-TGCGTGTCGTGGAGTC-3′

U6	RT:	5′-CGCTTCACGAATTTGCGTGTCAT-3′
	GSP:	5′-CGCTTCACGAATTTGCGTGTCAT-3′
	R:	5′-GCTTCGGCAGCACATATACTAAAAT-3′

β-actin	F:	5′-CACCCGCGAGTACAACCTTC-3′
	R:	5′-CCCATACCCACCATCACACC-3′

*Note:* F, forward primer; R, reversed primer; RT, reverse transcription primer.

Abbreviations: GSP, gene specific primer; Q-PCR, quantitative polymerase chain reaction.

## Data Availability

Data used in this study are available upon request from the corresponding author at dadonggene@sdutcm.edu.cn.

## Nomenclature

ADME: Absorption, distribution, metabolism, and excretion
DEGs: Differentially expressed genes
DL: Drug-likeness
EAU: Experimental autoimmune uveitis
GEO: Gene Expression Omnibus
IRBP: Interphotoreceptor retinoid-binding protein
LXD: Longdan Xiegan Decoction
miRNAs: MicroRNAs
OB: Oral bioavailability
OMIM: Online Mendelian inheritance in man
PPIs: Protein–protein interactions
TCM: Traditional Chinese medicine
TCMSP: Traditional Chinese Medicine Database and Analysis Platform
TLC: Thin-layer chromatography.

## Supporting Information

Supporting Information 1 Table S1: Criteria of the degree of inflammation.

Supporting Information 2 Table S2: Chemical information for the compounds of LXD.

Supporting Information 3 Figure S1: Original PPI network of drug-disease crossover genes.

Supporting Information 4 Figure S2: PPI network consisted of 168 hub genes.

Supporting Information 5 Table S3: Modular analysis of crossover target genes between active ingredients and uveitis-related targets in the PPI network.
