# Supplementary material for: Restoration of Immune Homeostasis: The Role of miR-30b-5p and Notch Signaling in Uveitis After Treatment With Longdan Xiegan Decoction
Source: Mediators Inflamm. 2025 Aug 28;2025:8824838. doi: 10.1155/mi/8824838 (PMC12411047; doi:10.1155/mi/8824838)
Supplement: Supporting Information 1 — Table S1: Criteria of the degree of inflammation. [file 8824838.f1.docx]

Supplement Table 1 Criteria of the degree of inflammation

| Score | Ocular inflammation |
| --- | --- |
| 0 | No disease |
| 1 | The iris was slightly dilated and the pupillary structure was abnormal |
| 2 | The iris was hyperemic, the pupil dilatation was limited, and the anterior chamber was slightly cloudy |
| 3 | The iris was heavily congested, the anterior chamber was moderately cloudy, and pupils were still visible |
| 4 | The iris was seriously congested, the anterior chamber was seriously cloudy and empyemic, and the pupil was closed |
