# Supplementary material for: Restoration of Immune Homeostasis: The Role of miR-30b-5p and Notch Signaling in Uveitis After Treatment With Longdan Xiegan Decoction
Source: Mediators Inflamm. 2025 Aug 28;2025:8824838. doi: 10.1155/mi/8824838 (PMC12411047; doi:10.1155/mi/8824838)
Supplement: Supporting Information 2 — Table S2: Chemical information for the compounds of LXD. [file 8824838.f2.docx]

Supplement Table 2 Chemical information for the compounds of LXD

| Medicinal herbs | Compounds | TCMSP Mol ID | PubChem Compound CID | OB (%) | DL |
| --- | --- | --- | --- | --- | --- |
| Gentianae Radix Et Rhozima | Gentiopicroside | MOL000646 | 88708 | 22.98 | 0.39 |
|  | Amarogentin | MOL003140 | 115149 | 1.89 | 0.77 |
|  | Sovitexin |  |  |  |  |
|  | Leucanthoside | MOL003137 | 442659 | 32.12 | 0.78 |
|  | Gentiopicrin | MOL000646 | 88708 | 22.98 | 0.39 |
|  | 1-o-beta-d-glucopyranosylamplexin |  |  |  |  |
|  | Gentiatibetine | MOL000649 |  | 62.59 | 0.05 |
|  | Sesamin | MOL001558 | 72307 | 56.55 | 0.83 |
|  | Swertiamarin | MOL003166 | 374.38 | -1.29 | 0.25 |
|  | longipinene | MOL001132 | 520957 | 17.01 | 0.12 |
|  | Stigmasterol | MOL000449 | 5280794 | 43.83 | 0.76 |
|  | Gentisein | MOL003170 | 5281635 | 67.57 | 0.19 |
|  | Gentisin | MOL003152 | 5281636 | 64.06 | 0.21 |
|  | Wogonin | MOL000173 | 5281703 | 30.68 | 0.23 |
|  | Trilobatin |  |  |  |  |
|  | gentirigenic acid | MOL003143 | 44423055 | 38.78 | 0.78 |
| Scutellariae Radix | Wogonoside/Oroxindin | MOL013068 | 3084961 | 7.07 | 0.77 |
|  | Wogonin | MOL000173 | 5281703 | 30.68 | 0.23 |
|  | Skullcapflavone_II | MOL002927 | 124211 | 69.51 | 0.44 |
|  | Ent-Epicatechin | MOL000073 | 182232 | 48.96 | 0.24 |
|  | Beta-sitosterol/ sitosterol | MOL000359 | 12303645 | 36.91 | 0.75 |
|  | Norwogonin | MOL000525 | 5281674 | 39.4 | 0.21 |
|  | Panicolin | MOL002932 | 5320399 | 76.26 | 0.29 |
|  | Rivularin | MOL012266 | 13889022 | 37.94 | 0.37 |
|  | Baicalin | MOL002776 | 64982 | 40.12 | 0.75 |
|  | Campesterol | MOL012254 | 173183 | 37.58 | 0.71 |
|  | Campesteryl_ferulate | MOL002669 | 15056832 | 22.1 | 0.59 |
|  | Dihydroperilla_alcohol |  | 519954 |  |  |
|  | Moslosooflavone | MOL008206 | 188316 | 44.09 | 0.25 |
|  | Nothosmyrnol_ |  | 5314067 |  |  |
|  | Sucrose | MOL000842 | 5988 | 7.17 | 0.23 |
|  | Woodorien |  | 192694 |  |  |
|  | Stigmasterol | MOL000449 | 5280794 | 43.83 | 0.76 |
|  | Coptisine | MOL001458 | 72322 | 30.67 | 0.86 |
|  | 5,8,2'-Trihydroxy-7-methoxyflavone | MOL002908 | 156992 | 37.01 | 0.27 |
|  | 5,2'-Dihydroxy-6,7,8-trimethoxyflavone | MOL000552 | 159029 | 31.71 | 0.35 |
|  | Epiberberine | MOL002897 | 160876 | 43.09 | 0.78 |
|  | Salvigenin | MOL002915 | 161271 | 49.07 | 0.33 |
|  | Dihydrooroxylin | MOL002937 | 25721350 | 66.06 | 0.23 |
|  | Carthamidin | MOL002910 | 188308 |  |  |
|  | Eriodyctiol | MOL002914 | 373261 |  |  |
|  | Oroxindin | MOL013068 | 3084961 | 7.07 | 0.77 |
|  | Acacetin | MOL001689 | 5280442 | 34.97 | 0.24 |
|  | Baicalein | MOL002714 | 5281605 | 33.52 | 0.21 |
|  | Dihydrooroxylin_A | MOL002926 | 5316733 | 38.72 | 0.23 |
|  | Oroxylin_A | MOL002928 | 5320315 | 34.97 | 0.24 |
|  | 5,7,2',6'-Tetrahydroxyflavone | MOL002925 | 5321865 | 37.01 | 0.24 |
|  | 5,7,4'-trihydroxy-6-methoxyflavanone | MOL012245 | 26213330 | 36.63 | 0.27 |
|  | 5,7,4'-Trihydroxy-8-methoxyflavone | MOL002933 | 5322078 | 36.56 | 0.27 |
|  | Dihydrobaicalin | MOL002912 | 14135325 | 20.85 | 0.75 |
|  | 5,7,4'-trihydroxy-8_methoxyflavanone | MOL002933 | 5322078 | 36.56 | 0.27 |
|  | 5,2',6'-Trihydroxy-7,8-dimethoxyflavone | MOL002917 | 5322059 | 45.05 | 0.33 |
| Radix Bupleuri | Octalupine | MOL004628 | 73404 | 47.82 | 0.28 |
|  | Cubebin | MOL013187 | 117443 | 57.13 | 0.64 |
|  | Sainfuran | MOL004644 | 185034 | 79.91 | 0.23 |
|  | Kaempferol | MOL000422 | 5280863 | 41.88 | 0.24 |
|  | Alpha-spinasterol | MOL004718 | 5281331 | 42.98 | 0.76 |
|  | Saikosaponin a | MOL004635 | 167928 | 32.39 | 0.09 |
|  | Saikosaponin d | MOL004637 | 107793 |  |  |
|  | Saikosaponin b | MOL004636 | 9875547 | 5.94 | 0.13 |
|  | Saikosaponin c | MOL004701 | 167927 | 5.12 | 0.05 |
| Alisma Orientale (Sam.) Juz | Sulfoorientalol_A | MOL000844 | 100942758 | 28.14 | 0.16 |
|  | Sulfoorientalol_B | MOL000845 | 100942914 | 11.3 | 0.18 |
|  | Sulfoorientalol_C | MOL000846 | 10470100 | 41.57 | 0.16 |
|  | Alisol_A | MOL000828 | 15558616 | 18.28 | 0.81 |
|  | Alisol_B | MOL000830 | 15558620 | 34.47 | 0.82 |
|  | Alisol_C | MOL000854 | 101306923 | 32.7 | 0.82 |
|  | Sitosterol | MOL000359 | 12303645 | 36.91 | 0.75 |
|  | Alisol_C_monoacetate | MOL000856 | 14036813 | 33.06 | 0.83 |
| Plantaginis Semen | Hypolaetin | MOL007819 | 5281648 | 33.24 | 0.28 |
|  | plantaginin_qt | MOL007836 | 5320623 | 54.04 | 0.24 |
|  | Dihydrotricetin | MOL007813 | 440836 | 58.12 | 0.28 |
|  | Dinatin | MOL001735 | 5281628 | 30.97 | 0.27 |
|  | Plantaginin | MOL007778 | 5320623 | 2.76 | 0.76 |
|  | Orobanchoside qt | MOL007835 | 6441894 | 55.99 | 0.82 |
|  | 3-Epioleanolic acid | MOL011390 | 7061302 | 16.07 | 0.76 |
| Angelicae Sinensis Radix | Ferulic_acid | MOL000360 | 445858 | 39.56 | 0.06 |
|  | (-)-alpha-Pinene | MOL000125 | 440968 | 46.25 | 0.05 |
|  | Beta-pinene | MOL000126 | 440967 | 44.84 | 0.05 |
|  | Ligustilide | MOL011782 | 5319022 | 23.5 | 0.07 |
|  | Adenine | MOL001788 | 190 | 62.81 | 0.03 |
|  | alpha-Terpinene | MOL000202 | 7462 | 33.02 | 0.02 |
| Rehmanniae Radix Praeparata | Catalpol | MOL002819 | 91520 | 5.07 | 0.44 |
|  | Dihydrocatalpol | MOL003700 | 5705531 | 3.58 | 0.44 |
|  | Adenosine | MOL001787 | 60961 |  |  |
|  | Coniferin | MOL002649 | 5280372 |  |  |
|  | Leonoside_F |  | 57325811 |  |  |
|  | Isoacteoside | MOL007795 | 6476333 | 1.52 | 0.66 |
|  | Uridine | MOL000059 | 6029 |  |  |
