# Supplementary figures and images for: Restoration of Immune Homeostasis: The Role of miR-30b-5p and Notch Signaling in Uveitis After Treatment With Longdan Xiegan Decoction
Source: Mediators Inflamm. 2025 Aug 28;2025:8824838. doi: 10.1155/mi/8824838 (PMC12411047; doi:10.1155/mi/8824838)

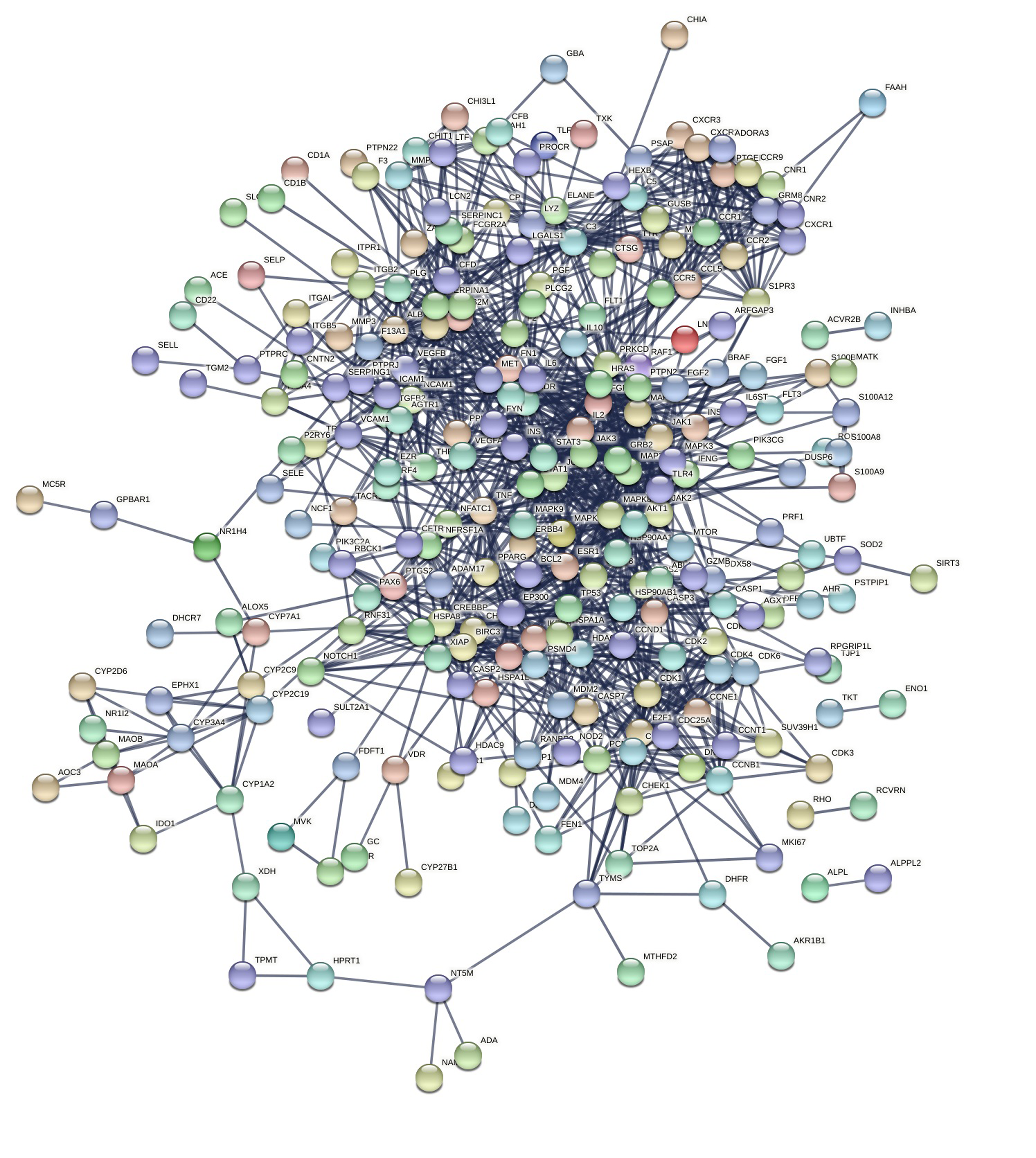

Supplement: Supporting Information 3 — Figure S1: Original PPI network of drug-disease crossover genes. [file 8824838.f3.tif]

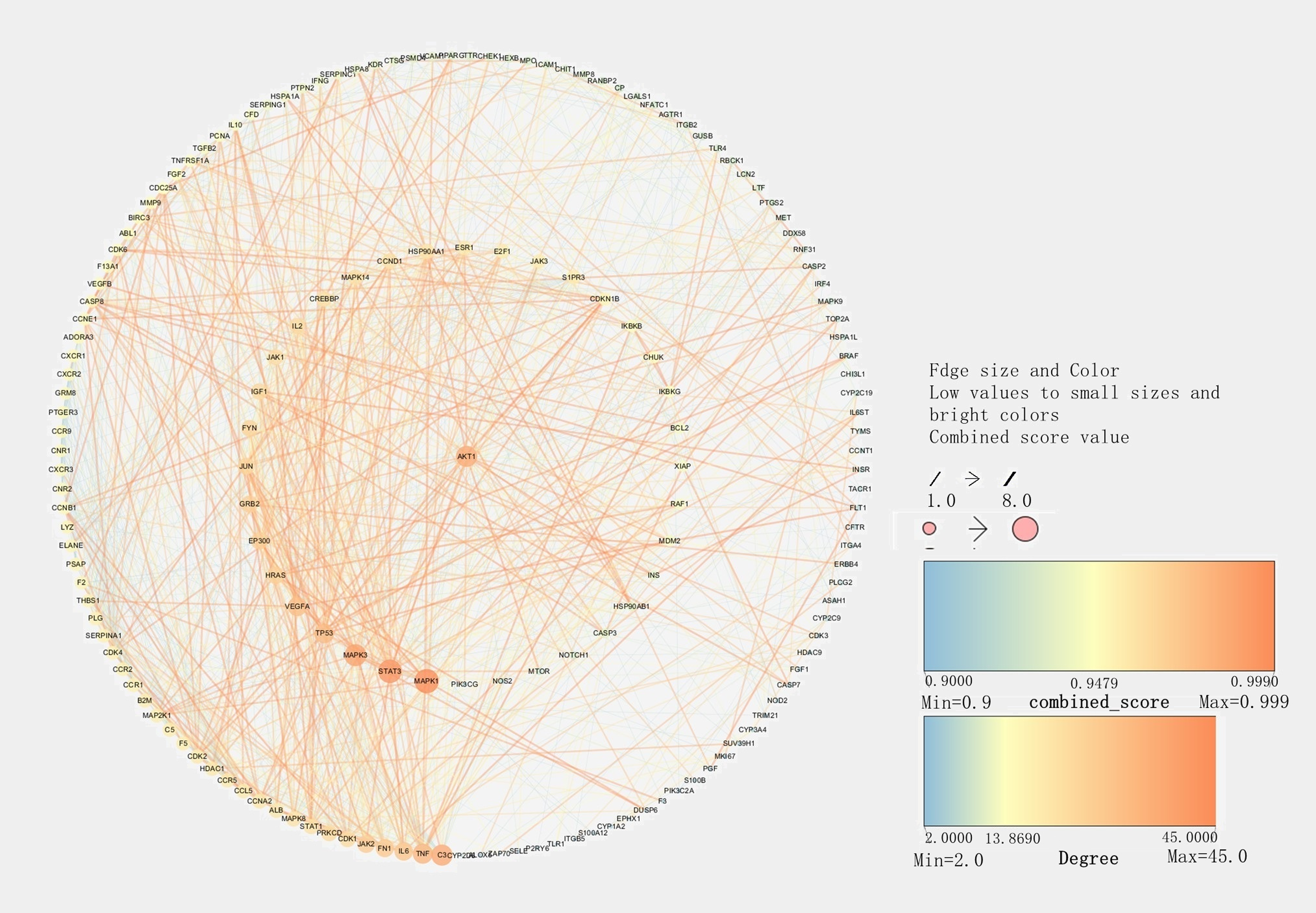

Supplement: Supporting Information 4 — Figure S2: PPI network consisted of 168 hub genes. [file 8824838.f4.tif]
