# Supplementary material for: Restoration of Immune Homeostasis: The Role of miR-30b-5p and Notch Signaling in Uveitis After Treatment With Longdan Xiegan Decoction
Source: Mediators Inflamm. 2025 Aug 28;2025:8824838. doi: 10.1155/mi/8824838 (PMC12411047; doi:10.1155/mi/8824838)
Supplement: Supporting Information 5 — Table S3: Modular analysis of crossover target genes between active ingredients and uveitis-related targets in the PPI network. [file 8824838.f5.docx]

Supplement Table 3 Modular analysis of crossover target genes between active ingredients and uveitis-related targets in the PPI network

| Module number | Score (Density*#Nodes) | Nodes | Edges | Node IDs |
| --- | --- | --- | --- | --- |
| 1 | 12.955 | 45 | 285 | S1PR3, PSAP, SERPINC1, ITGB2, CNR1, IL6, CCR9, SERPINA1, PTGER3, PLG, GRM8, CXCR2, GUSB, CCR1, CXCR1, CFD, CCR5, F5, CCR2, ELANE, C3, STAT3, PRKCD, C5, ADORA3, MAPK3, HEXB, CP, MPO, CTSG, FN1, TTR, LGALS1, ALB, MAPK1, AKT1, F2, LYZ, TNF, AGTR1, CNR2, JUN, CCL5, CXCR3, IL1 |
| 2 | 12.898 | 119 | 761 | SERPINC1, IL6, SUV39H1, B2M, S100A12, RANBP2, CP, PPARG, STAT1, CDK1, TTR, CDC25A, LGALS1, TOP2A, IKBKG, CDK3, HDAC9, MKI67, IKBKB, HRAS, CHEK1, IL6ST, NFATC1, ESR1, PCNA, JAK3, MET, HSPA1A, TYMS, BCL2, PTPN2, INS, CCNT1, HSP90AB1, JUN, CCNB1, XIAP, IL10, CHUK, MTOR, PIK3CG, JAK1, HSP90AA1, NOS2, CASP3, INSR, NOTCH1, TLR1, TP53, STAT3, DDX58, EP300, MAPK3, CREBBP, RAF1, IL2, MAPK1, AKT1, S100B, F2, FLT1, KDR, FGF2, FGF1, ITGB2, GUSB, F3, PRKCD, HEXB, GRB2, HDAC1, MPO, MAPK8, CTSG, FYN, MAP2K1, E2F1, CCNA2, JAK2, CDK2, CDK6, BRAF, CCNE1, MDM2, TLR4, CDK4, MAPK14, TNF, CDKN1B, CASP7, CCND1, ABL1, NOD2, RBCK1, RNF31, ERBB4, PSMD4, TNFRSF1A, BIRC3, CASP8, CASP2, SELE, ELANE, PLCG2, LCN2, VCAM1, ICAM1, CHI3L1, TRIM21, DUSP6, MMP9, CHIT1, LYZ, MMP8, IFNG, LTF, ASAH1, MAPK9, PTGS2 |
| 3 | 12.491 | 58 | 356 | SERPINC1, ITGB2, IL6, SERPINA1, PLG, B2M, CFD, F5, F3, VEGFB, TGFB2, PRKCD, SERPING1, GRB2, F13A1, CP, HDAC1, THBS1, CTSG, FN1, TTR, FYN, LGALS1, MAP2K1, ALB, ITGA4, JAK2, VEGFA, BRAF, HRAS, ESR1, MET, PTPN2, TNF, INS, ABL1, JUN, HSP90AA1, INSR, IGF1, ELANE, TP53, STAT3, EP300, MAPK3, CREBBP, RAF1, VCAM1, MAPK1, AKT1, PGF, F2, MMP9, MMP8, FLT1, KDR, PTGS2, FGF2 |
| 4 | 7.746 | 64 | 244 | FGF1, ITGB2, B2M, PPARG, MAPK8, FYN, HDAC9, CHEK1, BRAF, HRAS, IL6ST, MDM2, NFATC1, ESR1, HSPA1A, MET, BCL2, MAPK14, PTPN2, HSPA1L, INS, CCNT1, HSP90AB1, NOD2, JUN, IL10, ERBB4, MTOR, PIK3CG, HSP90AA1, ITGB5, NOS2, CASP3, INSR, NOTCH1, TP53, PLCG2, STAT3, IRF4, EP300, MAPK3, LCN2, CREBBP, RAF1, VCAM1, ZAP70, ICAM1, CHI3L1, AKT1, TRIM21, HSPA8, DUSP6, S100B, F2, MMP9, CHIT1, FLT1, MMP8, LTF, KDR, ASAH1, MAPK9, PTGS2, FGF2 |
| 5 | 4.5 | 9 | 18 | ALOX5, CYP3A4, CYP1A2, CYP2D6, NOS2, CYP2C19, PTGS2, EPHX1, CYP2C9 |
| 6 | 4.312 | 33 | 69 | ERBB4, MTOR, FGF1, HSP90AA1, CASP2, INSR, S100A12, DDX58, IRF4, EP300, MAPK3, RANBP2, PPARG, HDAC9, HSPA8, DUSP6, S100B, MDM2, NFATC1, TACR1, HSPA1A, MET, PIK3C2A, BCL2, PTPN2, HSPA1L, INS, IFNG, CFTR, AGTR1, KDR, HSP90AB1, JUN |
| 7 | 4 | 5 | 8 | AGTR1, TACR1, PIK3C2A, P2RY6, CFTR |
| 8 | 3.6 | 6 | 9 | ITGA4, VCAM1, SELE, ITGB5, ITGB2, ICAM1 |
| 9 | 2.667 | 4 | 4 | CASP2, CASP3, CASP7, MDM2 |
| 10 | 2.667 | 10 | 12 | HSPA1A, FLT1, PLCG2, IFNG, PGF, KDR, HSP90AB1, HSP90AA1, RANBP2, IL10 |
